# Supplementary material for: Wolf diet and prey selection in the South-Eastern Carpathian Mountains, Romania
Source: PLoS One. 2019 Nov 21;14(11):e0225424. doi: 10.1371/journal.pone.0225424 (PMC6874069; doi:10.1371/journal.pone.0225424)
Supplement: S2 Table — (PDF) [file pone.0225424.s002.pdf]

S2 Table. The prey to wolf ratio in 1970 and 2012, calculated based on species abundances estimated at the national level by the responsible Romanian authorities.

Prey to wolf ratio	1970	2012
wild boar : wolf	11 : 1	26 : 1
red deer : wolf	18 : 1	14 : 1
roe deer : wolf	128 : 1	65 : 1
livestock : wolf	16174 : 1	6762 : 1

*Livestock includes sheep, goat, cattle, pig.

Sources

1. Popescu C.C. 1973, Bonitarea noilor fonduri de vanatoare din Romania. Analele ICAS. 1973;29(2):25-62.

3. Geacu S. The wolf populations (*Canis lupus* L., Mammalia, Carnivora) in Romania and the human impact over the last two centuries. Rev Roum Géogr/Rom Journ Geogr. 2009;53(2):219-231.

3. http://www.mmediu.ro/beta/wp-content/uploads/2013/02/2013-02-26-DB-Raport-II.doc
