# Supplementary material for: Wolf diet and prey selection in the South-Eastern Carpathian Mountains, Romania
Source: PLoS One. 2019 Nov 21;14(11):e0225424. doi: 10.1371/journal.pone.0225424 (PMC6874069; doi:10.1371/journal.pone.0225424)
Supplement: Attachment — Submitted filename: Mori et al. 2017.pdf [file pone.0225424.s003.pdf]

Eur J Wildl Res
DOI 10.1007/s10344-016-1060-7

SHORT COMMUNICATION

What does the wild boar mean to the wolf?

Emiliano Mori[1] · Ludovica Benatti[1] · Sandro Lovari[1] · Francesco Ferretti[1]

Received: 26 June 2016 / Revised: 21 September 2016 / Accepted: 30 November 2016
© Springer-Verlag Berlin Heidelberg 2016

Abstract Generalist predators are expected to shape their diets according to the local availability of prey species. In turn, the extent of consumption of a prey would be influenced by the number of alternative prey species. We have tested this prediction by considering the wild boar and the grey wolf: two widespread species whose distribution ranges overlap largely in Southern Europe, e.g. in Italy. We have reviewed 16 studies from a total of 21 study areas, to assess whether the absolute frequency of occurrence of wild boar in the wolf diet was influenced by (*i*) occurrence of the other ungulate species in diet and (*ii*) the number of available ungulate species. Wild boar turned out to be the main prey of the wolf (49% occurrence, on average), followed by roe deer (24%) and livestock (18%). Occurrence of wild boar in the wolf diet decreased with increasing usage of roe deer, livestock, and to a lower extent, chamois and red deer. The number of prey species did not influence the occurrence of wild boar in the wolf diet. The wild boar is a gregarious, noisy and often locally abundant ungulate, thus easily detectable, to a predator. In turn, the extent of predation on this ungulate may not be influenced so much by the availability of other potential prey. Heavy artificial reductions of wild boar numbers, e.g. through numerical control, may concentrate predation by wolves on alternative prey (e.g. roe deer) and/or livestock, thus increasing conflicts with human activities.

✉ Sandro Lovari
lovari@unisi.it

[1] Research Unit of Behavioural Ecology, Ethology and Wildlife Management - Department of Life Sciences, University of Siena, Via P.A. Mattioli 4, 53100 Siena, Italy

Keywords Large carnivores · Feeding ecology · Human-wildlife conflict · Predator-prey relationships · Ungulates

Introduction

The composition of prey communities is expected to influence the diet of predators (e.g. Sinclair et al. 2003; Garrott et al. 2007; Baudrot et al. 2016). In turn, large carnivores play a pivotal role in ecosystems, as their action may generate cascading effects on lower trophic groups (e.g. Fortin et al. 2005; Beschta and Ripple 2009; Suraci et al. 2016). Feeding habits have evolved to maximise fitness and subject to constraints imposed by availability of food resources (MacArthur and Pianka 1966). Generalist predators are expected to shape their diet according to the local availability of prey species (Terraube et al. 2014; Baudrot et al. 2016).

The wild boar *Sus scrofa* is the most widespread ungulate in the world, favoured by its ecological plasticity, popularity as a game species and particularly high reproductive rate (Barrios-Garcia and Ballari 2012; Massei et al. 2014). In Europe, its main predator is the grey wolf *Canis lupus* (Newsome et al. 2016), a generalist carnivore (e.g., Mech 1970; Okarma 1997; Davis et al. 2012) which feeds mainly on meso-large mammals, especially ungulates (Meriggi and Lovari 1996; Meriggi et al. 2011; Newsome et al. 2016). In the last few decades, because of reintroductions and improved management (Apollonio et al. 2010), numbers of wild ungulates have increased all over Europe and have been the main determinants of range expansion and population growth of the wolf (Okarma 1997; Chapron et al. 2014; Galaverni et al. 2015). When wild prey is available, the wolf tends to prefer it to livestock (Meriggi and Lovari 1996). In particular, the wild boar is an important prey in Mediterranean countries, with variable frequencies of occurrence across areas (e.g. Meriggi et al. 1996; Barja 2009; Bassi

Published online: 09 December 2016

et al. 2012). Abundance of wild boar in the diet of wolves should lead to a decreased predation on livestock (Meriggi et al. 2011). However, Meriggi and Lovari (1996) reported that the presence of several wild ungulate species is necessary to reduce predation pressure on livestock. Thus, it is unclear whether the use of wild boar could be influenced by the richness of the prey community, as well as by livestock availability.

In our review, we have assessed the importance of wild boar for the wolf diet in Italy. In particular, we have evaluated whether the occurrence of wild boar in the diet of the wolf could depend on number of potential prey species, i.e. large ungulates. We predicted that the importance of wild boar in the wolf diet would decrease when the spectrum of potential prey species increases.

Materials and methods

We have reviewed information from eight published papers and eight grey' sources (e.g. dissertations and technical reports) on food habits of wolves in Italy, estimated through scat analyses (Fig. 1). There are indications that shifts in diet of large predators are not season dependent (e.g. wolf: Patalano and Lovari 1993; Iberian lynx: Ferreras et al. 2010; common

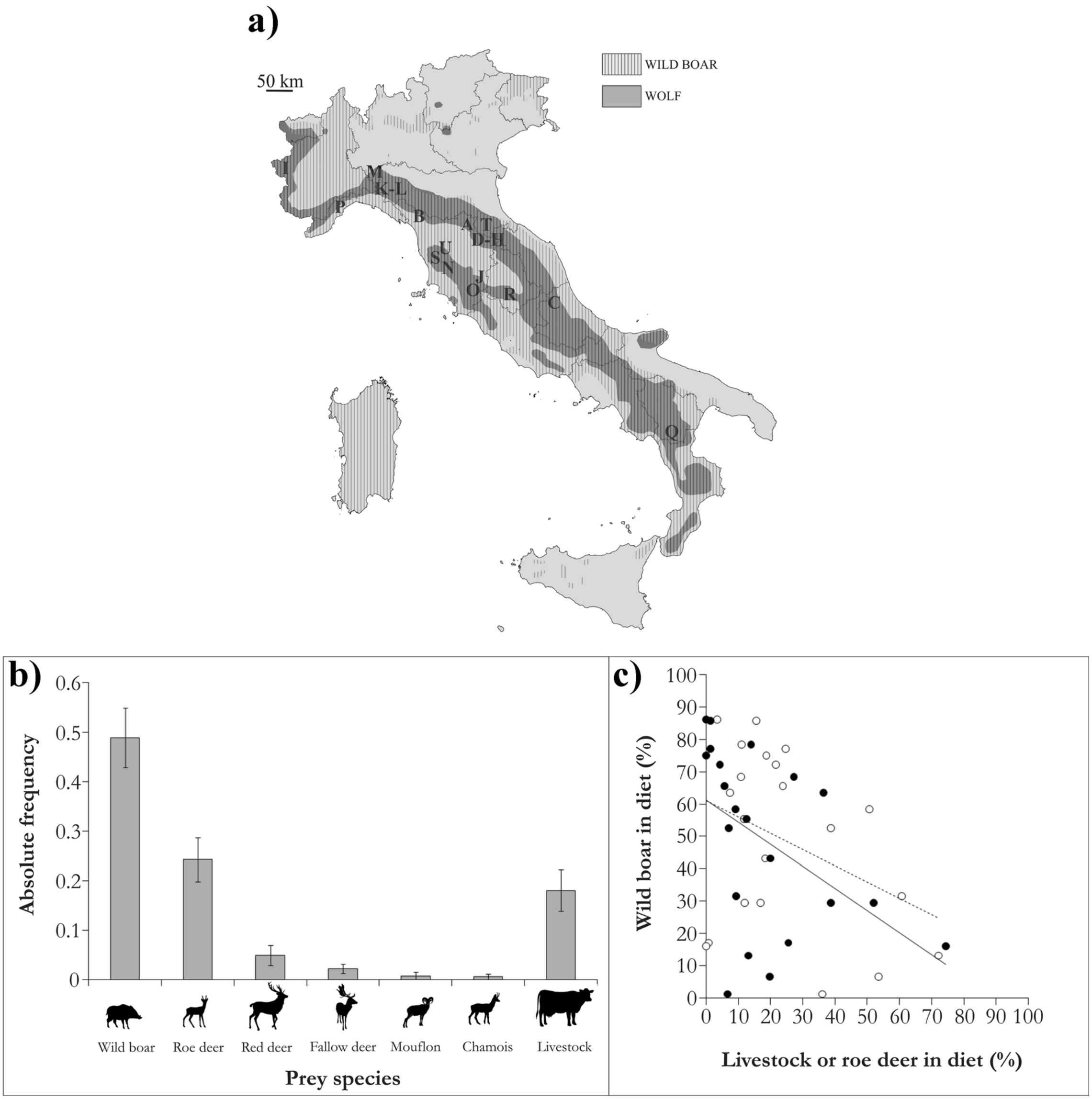

Fig. 1 **a** Distribution of study areas: *A* Mattioli et al. 1995; *B* Ciucci et al. 1996; *C* Patalano 2003; *D–H* Mattioli et al. 2004; *I* Gazzola et al. 2005; *J* Bargagli 2006; *K–L* Milanesi et al. 2012; *M* Meriggi et al. 2015; *N* Mori unpublished 2010–2015; *O* Mori unpublished 2013; *P* Brielli 2011; *Q* Borrelli 2010; *R* Vercillo and Ragni 2012; *S* Benatti 2015; *T* Lovari et al. 1995; *U* Boitani and Ciucci 1996. Distribution map of wolf and wild boar are taken from Lovari and Riga (2016) **b** absolute frequency of ungulate prey in the diet of the wolf and **c** absolute frequency of occurrence of wild boar in the diet of the wolf in relation to abs. frequency of occurrence of livestock (*empty circles*, *dotted line*) and roe deer (*black circles*, *solid line*)

and snow leopards: Lovari et al. 2013). For the wolf, Newsome et al. (2016, Appendix S5) concluded that the season is not a significant source of bias in assessment of diet.

We collated data on (*i*) absolute frequency of wild boar in the diet of wolves (i.e. number of scats with occurrence of wild boar/number of analysed scats, to avoid the inter-dependence bias of relative frequencies, see also Meriggi and Lovari 1996) and (*ii*) number of potential meso-large prey (i.e. ungulates: wild boar, roe deer *Capreolus capreolus*, red deer *Cervus elaphus*, fallow deer *Dama dama*, both species of chamois *Rupicapra rupicapra* and *Rupicapra pyrenaica*, mouflon *Ovis aries*, small livestock: sheep/goat, large livestock: cattle/equid). Mean absolute frequencies of each ungulate species were calculated across study areas (n = 21, from 16 sources). In 10 out of 21 study areas, the general category livestock' has been used. In turn, we calculated the absolute frequency of livestock, pooling together data relevant to single species, when available.

We used general linear models to assess relationships between the absolute frequency of wild boar in the diet of wolves (response variable) and the composition of the assembly of potential prey (i.e. the number of species), as well as the frequency of occurrence of other prey in diet (predictor variables). We detected collinearity between frequencies of occurrence of red deer and chamois in the wolf diet (correlation coefficient: r = 0.78); in turn, we considered the frequency of red deer only, because this prey species was comparatively more important for the wolf (see Fig. 1). Model selection was conducted through the 'MuMIn' package (Bartòn 2012) of the R software, fitting all possible models (n = 26). We used the Akaike's Information Criterion corrected for small sample sizes (AIC_c). Models were selected for inference if they had $\Delta AIC_c \leq 2$ units (Burnham and Anderson 1998) and if their AIC_c was lower than that of any simpler, nested alternative.

Results and discussion

The wild boar was the staple of the wolf diet (abs. frequency, mean ± standard error: 0.49 ± 0.06, occurring in all the studies), followed by roe deer (0.24 ± 0.04, found in 95.2% studies) and livestock (0.18 ± 0.04, found in 90.6% studies) (Fig. 1). Other ungulates were never the main prey of the wolf, but for the red deer in the upper Susa Valley (Gazzola et al. 2005), where wild boar presence was negligible (Gazzola et al. 2007). Fallow deer was reported as a prey in 42.9% studies, red deer in 33.3%, whereas both mouflon and chamois occurred in 9.5% studies each. Only the best model was retained for inference, including the effects of frequencies of roe deer, livestock and red deer (K = 5, log-likelihood = −87.500, AICc = 189.0, ΔAICc = 0.000, weight = 1.000). Thus, in contrast to our prediction, models did not support any effect of the number of potential ungulate prey species on the absolute frequency of wild boar in the wolf diet, but occurrence of livestock, roe deer and, to a lesser extent, that of red deer decreased with increasing absolute frequency of wild boar in diet (Fig. 1; Table 1).

Since the 1970s, land use changes (e.g. urbanisation and countryside abandonment by humans), the establishment of new protected areas, the implementation of national and international laws regulating hunting, as well as translocations of wildlife, have enhanced the numerical increase and range expansion of herbivore species in most of Europe (Acevedo and Cassinello 2009; Carnevali et al. 2009). Differently from roe deer, the wild boar is gregarious and noisy while moving, making itself easily detectable because of grunts, pungent smell and squeals (Cahill et al. 2003; Massei et al. 2014). In Europe, in the last few decades, the wild boar has been facing a sharp increase in numbers and distribution range (Massei et al. 2014), raising management concerns (Barrios-Garcia and Ballari 2012). Intensive hunting pressure is known to alter the spatial behaviour and social grouping of this ungulate (Maillard and Fournier 1995; Keuling et al. 2008), increasing its clumped distribution within wooded and protected areas (Tolon et al. 2009; Scillitani et al. 2010). Furthermore, post-natal body growth occurs more slowly in wild boar (Gaillard et al. 1992; Ježek et al. 2011) with respect to roe deer (Portier et al. 2000), the second most popular prey species for the wolf in the study areas covered by our review (Fig. 1). Most wild boar groups include a high number of juveniles, i.e. subadult individuals and piglets (e.g. Dardaillon 1988; Fernàndez-Llario et al. 1996), which fall well within the optimal prey size for the wolf (Gazzola et al. 2005).

Fallow deer (Gilbert 1968) and mouflons (Bon et al. 1990; Le Pendu et al. 1995) also live in medium-large sized groups, even where hunting pressure occurs (Svensson 2012), but these species, allochthonous in Europe, were much less common than wild boar in the areas of our review (i.e. always <5% in absolute presence for mouflon, up to 6% for fallow deer Carnevali et al. 2009; Benatti 2015). Several methodological flaws are inherent to most food habit studies, e.g. relating scat content to consumed prey and prey availability (Putman 1984), in turn even more so to reviews which have to rely on results obtained by other researchers. The wild boar is a prey twice as abundant as the roe deer (the second prey) in the

Table 1 Variables influencing the absolute frequency of occurrence of wild boar in the diet of the wolf in Italy estimated through general linear models; summary of the selected model (Multiple r^2 = 0.663; Adjusted r^2 = 0.604)

Predictor	*B*	s.e.	*p*
Intercept	94.522	8.835	<0.001
Livestock	−1.087	0.221	<0.001
Roe deer	−0.873	0.205	<0.001

wolf diet (Fig. 1). Therefore, even allowing for some approximation, we suggest that the wild boar makes an ideal prey species for the wolf.

Wild boar are often subjected to control operations to reduce strongly their density (e.g. Barrios-Garcia and Ballari 2012; Massei et al. 2014). One may speculate that a heavy artificial reduction of their local numbers, i.e. more than 50–70% over large areas (as several regional authorities have advocated recently through the media, in Italy), would deprive the wolf of its main prey species, thus intensifying predation on roe deer, a valued game animal, and livestock, in turn exacerbating the human-wolf conflict. Preventive actions to limit damage to agriculture and to livestock (e.g. appropriate fences, trained shepherd dogs, pragmatic legislation on wolf management), as well as different intensities of control of wild boar numbers in relation to ecological/agricultural vocation of areas, may be alternative/complementary measures to reduce human-wildlife conflicts.

Acknowledgements Thanks are due to Nicola Salomone and, in particular, to Marianna Patalano, who kindly provided us with their unpublished data on the diet of wolf. An anonymous reviewer and Alberto Meriggi improved our first draft with their comments.

References

Acevedo P, Cassinello J (2009) Human-induced range expansion of wild ungulates causes niche overlap between previously allopatric species: reed deer and Iberian ibex in mountainous regions of southern Spain. Ann Zool Fennici 46:39–50

Apollonio M, Andersen R, Putman R (eds) (2010) European ungulates and their management in the twenty-first century. Cambridge University Press, Cambridge

Bargagli L (2006) Analisi alimentare del lupo sul Monte Amiata. In: Lovari S, Sangiuliano A (ed.) Il lupo sul Monte Amiata. Comunità Montana Amiata Grosseto, Ministero dell'Ambiente, Ambiente, Arcidosso e Roma, Italy: 73–98

Barja I (2009) Prey and prey-age preference by the Iberian wolf *Canis lupus signatus* in a multiple-prey ecosystem. Wildl Biol 15:147–154

Barrios-Garcia MN, Ballari SA (2012) Impact of wild boar (*Sus scrofa*) in its introduced and native range: a review. Biol Inv 14:2283–2300

Bartòn K (2012) Package "MuMIn: Multi-model inference" for R, R Package Version 1.6.6 (http://CRAN.R-project.org/package=MuMIn). Downloaded on 30th May 2016

Bassi E, Donaggio E, Marcon A, Scandura M, Apollonio M (2012) Trophic niche overlap and wild ungulate consumption by red fox and wolf in a mountain area in Italy. Mamm Biol 77:369–376

Baudrot V, Perasso A, Fritsch C, Giraudoux P, Raoul F (2016) The adaptation of generalist predators' diet in a multi-prey context: insights from new functional responses. Ecology DOI. doi:10.1890/15-0427.1

Benatti L (2015) Variazioni geografiche dell'alimentazione del lupo *Canis lupus* L., 1758 in Italia. Tesi di Laurea in Scienze Biologiche, Dipartimento di Scienze della Vita, Università degli Studi di Siena, Italy

Beschta RL, Ripple WJ (2009) Large predators and trophic cascades in terrestrial ecosystems of the western United States. Biol Cons 142: 2401–2414

Boitani L, Ciucci P (1996) Programma di ricerca e gestione del lupo in Toscana. Relazione all'Amministrazione Regionale Toscana, Firenze

Bon R, Gonzalez G, Im S, Badia J (1990) Seasonal grouping in female mouflons in relation to food availability. Ethology 86:224–236

Borrelli S (2010) La dieta del lupo (*Canis lupus*) nel massiccio del Pollino. Tesi di laurea in Scienze Naturali, Università della Calabria, Italy

Brielli N (2011) La dieta del lupo (*Canis lupus* L, 1758) in Liguria in relazione all'abbondanza dei grandi erbivori domestici e selvatici. Tesi di laurea in Scienze Matematiche, Fisiche e Naturali, Università degli studi di Pavia, Italy

Burnham KP, Anderson DR (1998) Model selection and inference: a practical information—theoretic approach. Springer-Verlag, New York

Cahill S, Llimona F, Gràcia J (2003) Spacing and nocturnal activity of wild boar *Sus scrofa* in a Mediterranean metropolitan park. Wildl Biol 9:3–13

Carnevali L, Pedrotti L, Riga F, Toso S (2009) Banca Dati Ungulati: status, distribuzione, consistenza, gestione e prelievo venatorio delle popolazioni di Ungulati in Italia. Rapporto 2001-2005. Biol Cons Fauna 117:1–168

Chapron G, Kaczensky P, Linnel JDC, von Arx M, Huber D, Andrèn H, Lòpez-Bao JV, Adamec M, Alvares F, Anders O, Balčiauskas L, Balys V, Bedö P, Bego F, Blanco JC, Breitenmoser U, Brøseth H, Bufka L, Bunikyte R, Ciucci P, Dutsov A, Engleder T, Fuxjäger C, Groff C, Holmala K, Hoxha B, Iliopoulos Y, Ionescu O, Jeremić J, Jerina K, Kluth G, Knauer F, Kojola I, Kos I, Krofel M, Kubala J, Kunovac S, Kusak J, Kutal M, Liberg O, Majić A, Männil P, Mertzanis Y, Myslayek RW, Nowak S, Odden J, Ozolins J, Palomero G, Paunović M, Persson J, Potočnik H, Quenette PY, Rauer G, Reinhardt I, Rigg R, Ryser A, Salvatori V, Skrbinšek T, Stojanov A, Swenson JE, Szemethy L, Trajçe A, Tsingarska-Sedefcheva E, Váňa M, Veeroja R, Wabakken P, Wölfl M, Wölfl S, Zimmermann F, Zlatanova D, Boitani L (2014) Recovery of large carnivores in Europe's modern human-dominated landscapes. Science 346:1517–1519

Ciucci P, Boitani L, Raganella Pelliccioni E, Rocco M, Guy I (1996) A comparison of scat-analysis method to assess the diet of the wolf *Canis lupus*. Wildl Biol 2:37–48

Dardaillon M (1988) Wild boar social groupings and their seasonal changes in the Camargue, southern France. Z Säugetierkunde 53: 22–30

Davis ML, Stephens PA, Willis SG, Bassi E, Marcon A, Donaggio E, Capitani C, Apollonio M (2012) Prey selection by an apex predator: the importance of sampling uncertainty. PlosONE 7:e47894

Fernàndez-Llario P, Carranza J, Hidalgo de Trucios SJ (1996) Social organization of the wild boar (*Sus scrofa*) in Doñana National Park. Miscellània Zoològica 19:9–18

Ferreras P, Rodriguez A, Palomares F, Delibes M (2010) The difficult recovery of a critically endangered cat. In: Macdonald DW, Loveridge AJ (eds) Biology and conservation of wild felids. Oxford University Press, Oxford, pp. 507–520

Fortin D, Beyer HL, Boyce MS, Smith DW, Duchesne T, Mao JS (2005) Wolves influence elk movements: behavior shapes a trophic cascade in Yellowstone National Park. Ecology 86:1320–1330

Gaillard JM, Pontier D, Brandt S, Jullien JM, Allain D (1992) Sex differentiation in postnatal growth curve: a test in wild boar population. Oecologia 90:167–171

Galaverni M, Caniglia R, Fabbri E, Milanesi P, Randi E (2015) One, no one, or one thousand: how many wolves are there currently in Italy? Mamm Res 61:13–24

Garrott RA, Bruggeman JE, Becker MS, Kalinowski ST, White PJ (2007) Evaluating prey switching in wolf-ungulate systems. Ecol Appl 17: 1588–1597

Gazzola A, Bertelli I, Avanzinelli E, Tolosano A, Bertotto P, Apollonio M (2005) Predation by wolves (*Canis lupus*) on wild and domestic ungulates of the western alps. Ital J Zool 266:205–213

Gazzola A, Avanzinelli E, Bertelli I, Tolosano A, Bertotto P, Musso R, Apollonio M (2007) The role of the wolf in shaping a multi-species ungulate community in Italian western alps. Ital J Zool 74:297–307

Gilbert BK (1968) Development of social behaviour in the fallow deer (*Dama dama*). Z Tierpsychol 25:868–876

Ježek M, Štípek K, Kušta T, Červený J, Vícha J (2011) Reproductive and morphometric characteristics of wild boar in the Czech Republic. J Forest Sci 57:285–292

Keuling O, Stier N, Roth M (2008) How does hunting influence activity and spatial usage in wild boar *Sus scrofa* L.? Eur J Wildl Res 54:729–737

Le Pendu Y, Briedermann L, Gerard JF, Maublanc ML (1995) Inter-individual associations and social structure of a mouflon population (*Ovis orientalis musimon*). Behav Proc 34:67–80

Lovari C, Siemoni N, Castellani F, Centofanti E, Fasoli G (1995) Studio sulla biologia del lupo in rapporto alla presenza di ungulati selvatici e domestici nel Parco. D.R.E.A.M ITALIA, Pistoia

Lovari S, Riga F (2016) Manuale di gestione della fauna. Greentime publishers, Bologna

Lovari S, Minder I, Ferretti F, Mucci N, Randi E, Pellizzi B (2013) Common and snow leopards share prey, but not habitats: competition avoidance by large predators? J Zool (Lond) 291:127–135

MacArthur RH, Pianka EL (1966) On optimal time use of a patchy environment. Am Nat 100:603–609

Maillard D, Fournier P (1995) Effects of shooting with hounds on size of resting range of wild boar (*Sus scrofa* L.) groups in Mediterranean habitats. Ibex J Mountain Ecol 3:102–107

Massei G, Kindberg J, Licoppe A, Gačic D, Šprem N, Kamler J, Baubet E, Hohmann U, Monaco A, Ozoliņš J, Cellina S, Podgòrski T, Fonseca C, Markov N, Pokorny B, Rosell C, Nàhlik A (2014) Wild boar populations up, number of hunters down? A review of trends and implication for Europe. Pest Manag Sci 71:492–500

Mattioli L, Apollonio M, Mazzarone V, Centofanti E (1995) Wolf food habits and wild ungulate availability in the Foreste Casentinesi National Park, Italy. Acta Theriol 40:387–402

Mattioli L, Capitani C, Avanzinelli E, Bertelli I, Gazzola A, Apollonio M (2004) Predation by wolf (*Canis lupus*) on roe deer (*Capreolus capreolus*) in north-eastern Apennines. Ital J Zool 264:1–10

Mech LD (1970) The wolf: the ecology and behavior of an endangered species. The Natural History Press, New York

Meriggi A, Lovari S (1996) A review of wolf predation in southern Europe: does the wolf prefer wild prey to livestock? J Appl Ecol 33:1561–1571

Meriggi A, Brangi A, Matteucci C, Sacchi O (1996) The feeding habits of wolves in relation to large prey availability in northern Italy. Ecography 19:287–295

Meriggi A, Brangi A, Schenone L, Signorelli D, Milanesi P (2011) Changes of wolf (*Canis lupus*) diet in Italy in relation to the increase of wild ungulate abundance. Ethol Ecol Evol 23:195–210

Meriggi A, Dagradi V, Dondina O, Perversi M, Milanesi P, Lombardini M, Raviglione S, Repossi A (2015) Short-term responses of wolf feeding habits to changes of wild and domestic ungulate abundance in northern Italy. Ethol Ecol Evol 27:1–23

Milanesi P, Meriggi A, Merli E (2012) Selection of wild ungulates by wolf *Canis lupus* (L. 1758) in an area of the northern Appennines (North Italy). Ethol Ecol Evol 24:81–96

Newsome TM, Boitani L, Chapron G, Ciucci P, Dickman CR, Dellinger JA, Lòpez-Bao JV, Peterson RO, Shores CR, Wirsing AJ, Ripple WJ (2016) Food habits of the world's grey wolves. Mammal Rev doi. doi:10.1111/mam.12067

Okarma H (1997) The trophic ecology of wolves and their predatory role in ungulate communities of forest ecosystems in Europe. Acta Theriol 40:335–387

Patalano M (2003) La predazione sul bestiame domestico e l'ecologia alimentare del lupo nel Parco Nazionale del Gran Sasso e Monti della Laga. Relazione finale Ente Autonomo Parco Nazionale Gran Sasso e Monti della Laga, Assergi (L'Aquila), Italy

Patalano M, Lovari S (1993) Food habits and trophic niche overlap of the wolf and the red fox in a Mediterranean mountain area. Rev Ecol (Terre Vie) 48:279–294

Portier C, Duncan P, Gaillard JM, Guillon N, Sempèrè AJ (2000) Growth of European roe deer: patterns and rates. Acta Theriol 45:87–94

Putman RJ (1984) Facts from faeces. Mammal Rev 14:79–97

Scillitani L, Monaco A, Toso S (2010) Do intensive drive hunts affect wild boar (*Sus scrofa*) spatial behaviour in Italy? Some evidences and management implications. Eur J Wildl Res 56:307–318

Sinclair ARE, Mduma S, Brashares JS (2003) Patterns of predation in a diverse predator-prey system. Nature 425:288–290

Suraci JP, Clinchy M, Dill LM, Roberts D, Zanette LY (2016) Fear of large carnivores causes a trophic cascade. Nat Comm 7:10698

Svensson E (2012) Drevjaktens sekundära effekter på dovhinden (*Dama dama*). [The secondary effect of drive hunts on female fallow deer (*Dama dama*)]. Student Report 405, Swedish University of Agricultural Sciences, Department of Animal Environment and Health, Ethology and Animal Welfare programme, Skara, Sweden

Terraube J, Guixè D, Arroyo B (2014) Diet composition and foraging success in generalist predators: are specialist individuals better foragers? Basic Appl Ecol 15:616–624

Tolon V, Dray S, Loison A, Zeileis A, Fischer C, Baubet E (2009) Responding to spatial and temporal variations in predation risk: space use of a game species in a changing landscape of fear. Can J Zool 87:1129–1137

Vercillo F, Ragni B (2012) The diet of wolf in Umbria from 1967 until today: a story about a predator. Hystrix It J Mamm supp. 2012: 191
