# Supplementary material for: Wolf diet and prey selection in the South-Eastern Carpathian Mountains, Romania
Source: PLoS One. 2019 Nov 21;14(11):e0225424. doi: 10.1371/journal.pone.0225424 (PMC6874069; doi:10.1371/journal.pone.0225424)
Supplement: Attachment — Submitted filename: Imbert et al. 2016 WOLF & LIVESTOCK.pdf [file pone.0225424.s004.pdf]

ResearchGate

See discussions, stats, and author profiles for this publication at: https://www.researchgate.net/publication/290788567

Why do wolves eat livestock?

Article *in* Biological Conservation · March 2016

DOI: 10.1016/j.biocon.2016.01.003

CITATIONS
2

READS
2,352

8 authors, including:

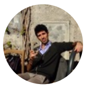

Romolo Caniglia
Institute for Environmental Protection and R…
68 PUBLICATIONS **505** CITATIONS

SEE PROFILE

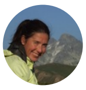

Elena Fabbri
Institute for Environmental Protection and R…
134 PUBLICATIONS **826** CITATIONS

SEE PROFILE

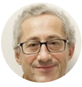

Ettore Randi
Institute for Environmental Protection and R…
474 PUBLICATIONS **7,659** CITATIONS

SEE PROFILE

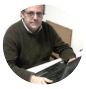

Meriggi Alberto
University of Pavia
141 PUBLICATIONS **1,140** CITATIONS

SEE PROFILE

Some of the authors of this publication are also working on these related projects:

Project Grouse Ecology on the Italian Alps View project

Project The Second European Breeding Bird Atlas (EBBA2) View project

All content following this page was uploaded by Ettore Randi on 01 February 2016.

The user has requested enhancement of the downloaded file.

Biological Conservation 195 (2016) 156–168

Contents lists available at ScienceDirect

Biological Conservation

journal homepage: www.elsevier.com/locate/bioc

Why do wolves eat livestock? Factors influencing wolf diet in northern Italy

Camille Imbert [a], Romolo Caniglia [b], Elena Fabbri [b], Pietro Milanesi [c], Ettore Randi [b,d], Matteo Serafini [c], Elisa Torretta [a], Alberto Meriggi [a,*]

[a] Dipartimento di Scienze della Terra e dell'Ambiente, Università di Pavia, Via A. Ferrata 1, 27100 Pavia, PV, Italy
[b] Laboratorio di Genetica, Istituto Superiore per la Protezione e Ricerca Ambientale (ISPRA), via Cà Fornacetta 9, 40064 Ozzano Emilia, BO, Italy
[c] Parco dell'Antola, La Torriglietta, Via N.S. Provvidenza 3, 16029 Torriglia, GE, Italy
[d] Department 18, Section of Environmental Engineering, Aalborg University, Sohngårdsholmsvej 57, 9000 Aalborg, Denmark

ARTICLE INFO

Article history:
Received 24 July 2015
Received in revised form 30 December 2015
Accepted 2 January 2016
Available online xxxx

Keywords:
Canis lupus
Scat analysis
Feeding ecology
Prey selection
Wolf–human conflicts

ABSTRACT

Thanks to protection by law and increasing habitat restoration, wolves (*Canis lupus*) are currently re-colonizing Europe from the surviving populations of Russia, the Balkan countries, Spain and Italy, raising the need to update conservation strategies. A major conservation issue is to restore connections and gene flow among fragmented populations, thus contrasting the deleterious consequences of isolation. Wolves in Italy are expanding from the Apennines towards the Alps, crossing the Ligurian Mountains (northern Italy) and establishing connections with the Dinaric populations. Wolf expansion is threatened by poaching and incidental killings, mainly due to livestock depredations and conflicts with shepherds, which could limit the establishment of stable populations. Aiming to find out the factors affecting the use of livestock by wolves, in this study we determined the composition of wolf diet in Liguria. We examined 1457 scats collected from 2008 to 2013. Individual scats were genotyped using a non-invasive genetic procedure, and their content was determined using microscopical analyses. Wolves in Liguria consumed mainly wild ungulates (64.4%; in particular wild boar *Sus scrofa* and roe deer *Capreolus capreolus*) and, to a lesser extent, livestock (26.3%; in particular goats *Capra hircus*). We modeled the consumption of livestock using environmental features, wild ungulate community diversity, husbandry characteristics and wolf social organization (stable packs or dispersing individuals). Wolf diet varied according to years and seasons with an overall decrease of livestock and an increase of wild ungulate consumption, but also between packs and dispersing individuals with greater livestock consumption for the latter. The presence of stable packs, instead of dispersing wolves, the adoption of prevention measures on pastures, roe deer abundance, and the percentage of deciduous woods, reduced predation on livestock. Thus, we suggest promoting wild ungulate expansion, the use of prevention tools in pastures, and supporting wolf pack establishment, avoiding lethal control and poaching, to mitigate conflicts between wolf conservation and husbandry.

Published by Elsevier B.V.

1. Introduction

The wolf (*Canis lupus*), because of its adaptability to different environments and its ability to re-colonize territories when no persecution occurs, has in just a few decades expanded its range in Europe (Balciauskas, 2008; Breitenmoser, 1998; Chapron et al., 2003, 2014). The Russian wolf population is the largest in Europe, supporting those of Baltic and North-European countries, and it is contiguous with the populations of Eastern Europe from which wolves began the re-colonization of Central Europe (Ansorge et al., 2006; Linnell et al., 2005). The Spanish wolf *Canis lupus signatus* (2200–2300 individuals) is slowly extending its distribution (Mech and Boitani, 2003).

Wolves greatly declined in Italy, surviving in two small isolated sub-populations confined to the southern and central part of the Apennines. At their nadir in the early seventies of the last century, wolves in Italy numbered about 100 individuals (Zimen and Boitani, 1975). Since the late eighties, wolves have shown a spontaneous rapid recovery, re-colonizing all the Apennines and reaching the western Italian and French Alps (Boitani, 2000; Breitenmoser, 1998; Fabbri et al., 2007; Marucco and McIntire, 2010; Valière et al., 2003).

The re-colonization of the Alps would be a fundamental step for wolf conservation in Italy and Central Europe as well (Genovesi, 2002). Moreover, the early and ongoing wolf expansion from the eastern

* Corresponding author.
E-mail addresses: cimbert@hotmail.fr (C. Imbert), romolo.caniglia@isprambiente.it (R. Caniglia), elena.fabbri@isprambiente.it (E. Fabbri), pietro.milanesi@unipv.it (P. Milanesi), ettore.randi@isprambiente.it (E. Randi), teos.teos@libero.it (M. Serafini), torretta.elisa@gmail.com (E. Torretta), meriggi@unipv.it, alberto.meriggi@unipv.it (A. Meriggi).

http://dx.doi.org/10.1016/j.biocon.2016.01.003
0006-3207/Published by Elsevier B.V.

Alps will predictably increase chances to originate mixed packs and increase the local genetic diversity as has been already described (Fabbri et al., 2014; Randi, 2011).

The sub-population of wolves inhabiting the Liguria region thus plays a crucial role in assuring the linkage between the wolves of central Italy and those of the Western Alps (Fabbri et al., 2007). If this link should break, the wolf population of the Western Alps would be isolated, perhaps failing to recolonize the remaining part of the Alps.

The distribution of wolves is usually determined by the abundance of its preys, environmental characteristics, and the risk associated with the presence of humans (Eggermann et al., 2011; Jędrzejewski et al., 2004; Massolo and Meriggi, 1998). This last point is the key problem of wolf conservation because wolves can have a dramatic impact on livestock breeding, affecting human attitudes that can lead to illegal killing, increasing the risk of extinction (Behdarvand et al., 2014; Kovařík et al., 2014).

The impact of wolves on livestock is different according to geographical region. In regions with a very low abundance of wild ungulates, as in Portugal and Greece, wolves feed mainly on livestock (Migli et al., 2005; Papageorgiou et al., 1994; Vos, 2000). On the other hand, in Germany attacks on livestock are rare because shepherds equip the pastures with electric fences to protect their herds and because the wild ungulate availability is high (Ansorge et al., 2006).

In other new-recolonizing areas such as France or North Italy, wild ungulates are the main prey of wolves, but the use of livestock is still noticeable (MEEDDAT–MAP, 2008; Meriggi et al., 2011; Milanesi et al., 2012).

Systematic research on wolf feeding ecology has been carried out since 1987 in the Ligurian Apennines. These studies showed an increasing use of wild ungulates in the time but also a medium–high use of livestock species as prey (Meriggi et al., 1991, 1996, 2011; Schenone et al., 2004). Consequently, wolf presence in Liguria, as well as in other areas of natural re-colonization, causes a conflict with human populations that perceive predator presence as a negative element that can compromise a poor rural economy. Thus, wolves suffer a high mortality mainly due to illegal killing and accidents. This situation makes the population vulnerable and actions aimed at a greater protection of the species are required.

Usually wolf populations are structured in stable packs and lone wolves; packs are formed by a pair of adults, by their offspring and other related individuals (i.e. the offspring of previous years), and sometime by adopted individuals, whereas lone wolves are erratic individuals that can temporarily establish in an area without packs. In general lone wolves are young dispersing from packs but they can also be adults moving far from their original pack because of pack disruption or break off for several causes (killing by humans, low prey availability and related increasing aggressiveness, natural death of the dominant pair) (Mech and Boitani, 2003). Packs are established in areas with high prey availability, because only a high availability of preferred prey can dampen the aggressiveness of the pack members and avoid pack disruption (Thurber and Peterson, 1993). Dispersing and erratic individuals use the areas without wolf packs that can be considered suboptimal habitats because of the low prey availability, high human disturbance, and possibly potential problems with local people (Fritts and Mech, 1981). Illegal killing can break the packs, increasing erratic wolves and reproductive pairs that can have a greater impact in particular on livestock rearing (Wielgus and Peebles, 2014).

The objective of the present study was to determine which factors influence wolf diet, in particular, the choice of livestock as prey, which is the first step to find solutions for wolf conservation. With this aim, we determined wolf diet, by analyses of scats collected in the whole Liguria region from 2008 to 2013. We highlighted the factors influencing it, i.e. years, seasons, ungulate abundance, and social structure of wolves (packs or dispersing individuals). Then we related livestock consumption to environmental features, wild ungulate abundance and diversity, husbandry characteristics, wolf grouping and habitat occupancy behavior (stable packs or dispersing individuals).

2. Material and methods

2.1. Study area

This research was carried out in the Liguria region, north Italy (44°30′16″, 8°24′10″). The study area spreads over 5343 km^2 including a part of the Northern Apennines and of the Western Italian Alps, until the border with France. The region is divided in four provinces, Imperia, Savona, Genoa and La Spezia, respectively from the western to the eastern part (Fig. 1). Altitude ranges from 0 to 2153 m a.s.l.; 36% of the area is between 0 and 400 m a.s.l., 35% between 400 and 800 m, 21% between 800 and 1200 m, and 8.5% more than 1200 m a.s.l. Forests cover 63.8% of the region (deciduous woods: 28.8%; conifer woods: 7.1%; mixed woods: 27.9%), pastures 6.2%, agricultural areas 17.1%, and urbanized areas 3.9%. Towns and villages, as well as farmlands, are concentrated on flat terrains, close to the coasts. The climate extends from Mediterranean on the coast to sub-oceanic in the mountains. The temperature extends from −2 °C in winter to 35 °C during summer. Mean annual precipitation ranges from 750 to 1250 mm in the west to 1350–1850 in the central and eastern part of the region. On the ridge of the mountains and in the upper part of the valleys, snow cover can reach more than one meter from November to April.

The wild ungulate community includes wild boar (*Sus scrofa*), widely distributed with high densities (21,500 individuals shot per year in Liguria, on average from 2007 to 2012), roe deer (*Capreolus capreolus*), abundant in particular in the central provinces (30.9 individuals per km^2 on average from 2009 to 2012). Fallow deer (*Dama dama*), introduced for hunting, is present in the provinces of Genoa and Savona (10.7 and 5.8 individuals per km^2 respectively). Chamois (*Rupicapra rupicapra*) is present only in the Maritime Alps (927 individuals counted on average from 2007 to 2012), while red deer (*Cervus elaphus*) and mouflon (*Ovis aries musimon*) are very rare in the study area (data from Wildlife Services of Imperia, Savona, Genoa and La Spezia).

This high availability of wild prey promoted a natural re-colonization of the region by wolves in the late eighties, starting from the provinces of Genoa and La Spezia (Meriggi et al., 1991, 1996, 2011; Schenone et al., 2004). Now the wolf is present in the four provinces with a minimum population of 58 individuals of which 21 distributed in 5 packs and 37 lone wolves, estimated by genetic analyses (see Results).

Livestock (15,000 cows and 33,900 sheep and goats) are free-grazing on pastures from April to October but the grazing period can be expanded or reduced depending on the weather. Pastures are often partly composed of shrubs and woodlots. Only few shepherds adopt prevention methods (i.e. nocturnal recovery, guardian dogs, and electric fences) to deter wolf attacks.

2.2. Data collection

We divided the study area in 64 isometric cells of 10 × 10 km, as a trade-off between the average territory size of the wolf in Italy (Ciucci et al., 1997; Corsi et al., 1999; Apollonio et al., 2004; Caniglia et al., 2014) and sampling feasibility. In each cell, we randomly chose an itinerary among the existing footpaths according to the Tessellation Stratified Sampling (TSS) method that permits a better distribution and representativeness of the random samples than a simple random design (Barabesi and Fattorini, 2013; Barabesi and Franceschi, 2011). We traced a total of 64 itineraries in the study area (total length = 287.6 km, mean ± SD = 4.5 ± 1.59 km, min. = 2.3, max. = 10.4) that were covered once a season (spring: March to May; summer: June to August; autumn: September to November; winter: December to February), from January 2008 to August 2013 searching for wolf scats and signs of wild ungulate presence (tracks, sightings, rooting,

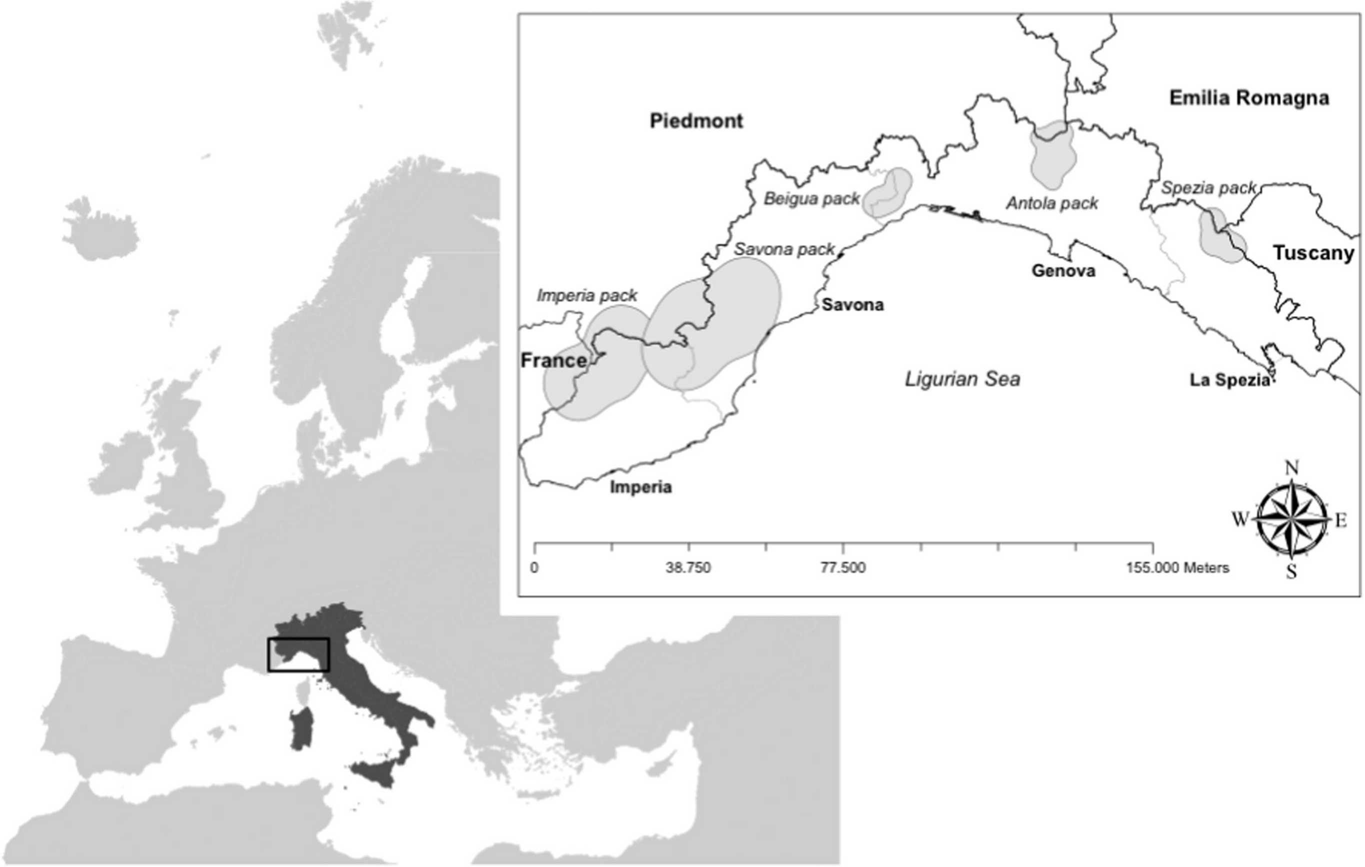

Fig. 1. Provinces of Liguria region and wolf pack territories.

rubbing, wallowing, resting sites, feeding and territorial marks). We identified wolf scats by the size, texture, shape, and their characteristic odor. All signs of presence were mapped and georeferred by a Garmin GPS.

We assessed wild ungulate abundance at transect level by computing an Index of Kilometric Abundance (IKA) for each species (number of found signs divided by the transect length, Meriggi et al., 1991, 1996, 2015; Milanesi et al., 2012). We estimated the abundance of livestock (cattle, sheep, goats, and horses) on pastures and defined the husbandry practices by direct interviews with shepherds and by the official data of Veterinary services of the four provinces.

Around each transect we defined a buffer zone corresponding to the potential hunting area of wolves. We used a width of 13 km, corresponding to the average travel distance of wolves during the night to go from dens or resting sites to hunting sites in Italy (Ciucci et al., 1997).

In each buffer, we measured from the Corine Land Cover III level and the Digital Elevation Models (DEM) 12 environmental variables concerning the land use, altitude, aspect, and slope using Arc GIS 9.0 software (Appendix A). Moreover, in each buffer we defined the husbandry variables: number of livestock heads, reared species, presence or absence of prevention methods, number of used pastures, average time past on pastures and the presence of the production "Cow-Calf line" consisting in cows giving birth alone, in the pasture and involving a great risk of predation by wolves on newborn calves (Dondina et al., 2014; Meriggi et al., 1991, 1996).

We also collected all claimed and verified cases of predation upon livestock during the study period, recording the preyed species and the exact location of the events.

2.3. Genetic analyses

From 2007 to 2013 we collected a total of 403 presumed wolf biological samples for genetic analyses. The genetic samples included 6 tissue and blood samples obtained from wolves found dead in the study area, 5 of urine, 2 hairs and 389 fresh scats containing cells of intestine epithelium. Small external portions of scats and clean tissue fragments were individually stored at −20 °C in 10 vials of 95% ethanol. Blood samples were stored at −20 °C in 2 vials of a Tris–sodium dodecyl sulfate buffer. DNA was automatically extracted using a MULTIPROBE IIEX Robotic Liquid Handling System (Perkin Elmer, Weiterstadt, Germany) and QIAGEN QIAmp DNA stool or DNeasy tissue extraction kits (Qiagen Inc., Hilden, Germany).

We identified individual genotypes for samples at 12 unlinked autosomal canine microsatellites (short tandem repeats [STR]): 7 dinucleotides (CPH2, CPH4, CPH5, CPH8, CPH12, C09.250, and C20.253) and 5 tetranucleotides (FH2004, FH2079, FH2088, FH2096, and FH2137), selected for their high polymorphism and reliable scorability for wolves and dogs (Caniglia et al., 2014).

We determined sex of samples using a polymerase chain reaction (PCR)–restriction fragment length polymorphism assay of diagnostic ZFX/ZFY gene sequences (Caniglia et al., 2012, 2013, 2014). We used a first panel of 6 STR to identify the genotypes with Hardy–Weinberg probability-of-identity (PID) among unrelated individuals, $PID = 8.2 \times 10^6$, and expected full-siblings, $PID_{sibs} = 7.3 \times 10^3$ (Mills et al., 2000; Waits et al., 2001) in the reference Italian wolves. We then used another panel of 6 STR, also selected for their polymorphism and reliable scorability, to increase the power of admixture and kinship analyses, decreasing the PID values to $PID = 7.7 \times 10^9$ and $PID_{sibs} = 3.1 \times 10^4$ (Caniglia et al., 2014). We identified maternal haplotypes by sequencing 350 base pairs of the mitochondrial DNA (mtDNA) control region, diagnostic for the haplotype W14, which is unique to the Italian wolf population, using primers L-Pro and H350 (Randi et al., 2000; Caniglia et al., 2014). We identified paternal haplotypes by typing 4 Y-linked microsatellites (Y-STR), MS34A, MS34B, MSY41A, and MS41B (Sundqvist et al., 2001), characterized by distinct allele frequencies in

dogs and wolves (Iacolina et al., 2010). We amplified autosomal and Y-linked STR loci in 7 multiplexed primer mixes using the QIAGEN Multiplex PCR Kit (Qiagen Inc.), a GeneAmp PCR System 9700 Thermal Cycler (Applied Biosystems, Foster City, California), and the following thermal profile: 94 °C for 15 min, 94 °C for 30 s, 57 °C for 90 s, 72 °C for 60 s (40 cycles for scat, urine, and hair samples, and 35 cycles for muscle and blood samples), followed by a final extension step of 72 °C for 10 min. We carried out amplifications in 10-μl volumes including 2 μl of DNA extraction solutions from scat, urine, and hair samples, 1 μl from muscle or blood samples (corresponding to approximately 20–40 ng of DNA), 5 μl of QIAGEN Multiplex PCR Kit, 1 μl of QIAGEN Q solution (Qiagen Inc.), 0.4 μM deoxynucleotide triphosphates, from 0.1 to 0.4 μl of 10 μM primer mix (forward and reverse), and RNase-free water up to the final volume. We amplified the mtDNA control region in a 10-μl PCR, including 1 or 2 μl of DNA solution, 0.3 pmol of the primers L-Pro and H350, using the following thermal profile: 94 °C for 2 min, 94 °C for 15 s, 55 °C for 15 s, 72 °C for 30 s (40 cycles), followed by a final extension of 72 °C for 5 min. PCR products were purified using exonuclease/shrimp alkaline phosphatase (Exo-Sap; Amersham, Freiburg, Germany) and sequenced in both directions using the Applied Biosystems Big Dye Terminator kit (Applied Biosystems, Foster City, California) with the following steps: 96 °C for 10 s, 55 °C for 5 s, and 60 °C for 4 min of final extension (25 cycles).

DNA from scat, urine, and hair samples was extracted, amplified, and genotyped in separate chambers reserved for low-template DNA samples, under sterile ultraviolet laminar flow hoods, following a multiple-tube protocol (Caniglia et al., 2012, 2013), including both negative and positive controls. We obtained genotypes from scat, urine, and hair samples replicating the analyses from 4 to 8 times, and from blood and muscle DNA replicating the analyses twice. DNA sequences and microsatellites were analyzed in a 3130XL ABI automated sequencer (Applied Biosystems), using the ABI software SEQSCAPE 2.5 for sequences, GENEMAPPER 4.0 for microsatellites (Applied Biosystems) (Caniglia et al., 2014) and GIMLET to control the good attribution of several samples to the same individual.

We assigned individual genotypes to their population of origin (wolves or dogs) using the Bayesian software STRUCTURE 2.3 (Falush et al., 2003). According to previous studies (Caniglia et al., 2014), the optimal number of populations was set at $K = 2$, the value that maximized the posterior probability of the data. At $K = 2$, we assessed the average proportion of membership (qi) of the sampled populations to the inferred clusters. Then we assigned genotypes to the Italian wolf or dog clusters at threshold $qi = 95$ (individual proportion of membership; Randi, 2008), or identified them as admixed if their qi values were intermediate.

We identified familiar relationships i.e. packs, using a maximum-likelihood approach (Caniglia et al., 2014) implemented in the software COLONY 2.0 (Wang and Santure, 2009). We selected all the genotypes that were sampled in restricted ranges (<100 km^2) at least 4 times and for periods longer than 24 months. We determined their spatial distributions by 95% kernel analysis, choosing band width using the least-squares cross-validation method (Kernohan et al., 2001; Seaman et al., 1999), using the ADEHABITATHR package for R (Calenge, 2006) and mapped them using ARCGIS 10.0. According to spatial overlaps, we split individuals into distinct groups that might correspond to packs, for which we performed parentage analyses. We considered as candidate parents of each group all the individuals sampled in the 1st year of sampling and more than 4 times in the same area and as candidate offspring all the individuals collected within the 95% kernel spatial distribution of each pack and in a surrounding buffer area of approximately 17-km radius from the kernel (see Caniglia et al., 2014). We ran COLONY with allele frequencies and PCR error rates as estimated from all the genotypes, assuming a 0.5 probability of including fathers and mothers in the candidate parental pairs.

2.4. Diet analysis

All the scats found on itineraries were preserved in PVC bags at −20 °C for 1 month, and then washed in water over two sieves with decreasing meshes (0.5–0.1 mm). We identified prey species from undigested remains: hair, bone, hoof, and claw (medium and large-sized mammals), hair and mandible (small mammals), seeds and leaves (fruits and plants). Moreover, hairs were washed in alcohol and observed with an optical microscope (Leica DM750) to identify the species from the characteristics of cortical scales, medulla, and root (Brunner and Coman, 1974; Debrot et al., 1982; De Marinis and Asprea, 2006; Teerink, 1991). We estimated the proportion of prey for each scat as they were eaten (Kruuk and Parish, 1981; Meriggi et al., 1991, 1996, 2015; Milanesi et al., 2012) and each prey species was assigned to a percent volumetric class: <1%; 1–5%; 6–25%; 26–50%; 51–75%; 76–95%; >95% that was converted in a final percent volume: 0.5%; 2.5%; 15.5%; 38%; 63%; 85.6% and 98% respectively. Prey species were grouped in six food categories (wild ungulates, livestock, small mammals, medium-sized mammals, fruits, grasses). We calculated the mean percent volume (MV%) and the percent frequency of occurrence (FO%) for each food category and species of wild and domestic ungulates. We determined the diet composition for two main seasons (grazing season: from April to October when livestock is on the pastures, and non-grazing season: from November to March), for each year of study, and for each itinerary (pooling the study years), for each pack, for pooled packs and for dispersing individuals.

2.5. Data analysis

We adapted the index provided by Massolo and Meriggi (1998) as a measure of the diversity of wild ungulate community; we used the IKA as a measure of abundance and 5 identical classes for all wild ungulate species. We divided the range of the IKA values for all species pooled to determine the class intervals for each season because the probability of detecting a track depends on the weather, mainly the presence of snow, mud, or leaves:

$$\mathrm{DI} = \frac{\Sigma \mathrm{A_i} * N}{\Sigma \mathrm{A_{i\,max}} * \mathrm{K}}$$

where A_i is the class of abundance of ith species, A_{imax} the class of maximum abundance of the ith species, N the number of species present for a transect, and K the maximum number of species. The index was calculated for each transect.

We defined pack territories by Kernel Analyses at 95% on GPS coordinates of genetic samples of wolves with parental ties. Scats within territories were considered to belong to the relative pack, and those outside the territories were assumed to belong to dispersing individuals. The scats localized on the overlap of two territories were not included in the analysis, because their origin was not identifiable with certainty.

To estimate the minimum number of scats necessary to assess the diet of wolves we used the Brillouin index (1956) (Hass, 2009; Milanesi et al., 2012; Meriggi et al., 2015):

$$\mathrm{H_b} = \frac{\ln \mathrm{N}! - \Sigma \ln \mathrm{n_i}!}{\mathrm{N}}$$

where $\mathrm{H_b}$ is the diversity of prey in the sample, N is the total number of single prey taxa in all samples, and n_i is the number of single prey taxa of the ith category. For each sample, a value of $\mathrm{H_b}$ was calculated and then re-sampled 1000 times by the bootstrap method to obtain the average values and 95% confidence intervals. We determined the adequacy of sample size by whether an asymptote was reached in the diversity curve and in the curve obtained from the incremental change in each $\mathrm{H_b}$ with the addition of two more samples.

We evaluated the significance of the differences in the diet between years and seasons by two-way non-parametric multivariate analysis of variance (NPMANOVA), and between packs and dispersing individuals by one-way NPMANOVA with permutation (10,000 replicates), using the Bonferroni correction of the p-value for pair-wise comparisons (Anderson, 2000, 2001). Annual, seasonal and pack-dispersing wolf variations of mean percent volumes of all categories and all ungulate species were verified by Kruskall–Wallis test with permutation (10,000 replicates).

Moreover we compared the observed and expected use of livestock species for each pack and for dispersing wolves by the Chi-square goodness-of-fit (Exact test) and Bonferroni's confidence interval analyses, testing the null hypothesis (H_0) of a use proportional to the availability (Manly et al., 2002). In this analysis we considered predation cases as use; in particular we calculated the expected frequencies from the availability of livestock (number of heads) in each pack territory and outside pack areas.

To identify the main factors affecting livestock consumption by wolves we carried out Multiple Regression Analyses (MRA) of MV% of domestic ungulates recorded for each transect vs. the variables measured in the buffers around the transects; only transects ($N = 34$) with at least 10 scats which corresponds, according to the Brillouin diversity index, to an incremental change of 5% for grazing season and 3% for non-grazing one, were included in the analyses. We identified all the possible subsets of uncorrelated ($P > 0.05$) predictor variables by calculating the correlation matrix (Pearson product moment coefficient) among habitat variables. For each subset, we performed MRA between MV% of livestock and transect variables. We obtained a number of models that were ranked by the information theoretic approach (Akaike, 1973). We computed the corrected value of Akaike information criterion (AICc) because the ratio sample/parameters was small (Burnham and Anderson, 2002), selecting the model with the lowest AICc as the best model and ranking the following ones by their differences from the lowest AICc (Δ_i). For the following analysis, we considered only models with $\Delta_i \leq 2$ (Best and Rayner, 2007; Burnham and Anderson, 2002). Furthermore, we measured the relative importance of models by their Akaike weights (w_i; Anderson et al., 2000, 2001). We followed the AIC statistical approach because it allows the comparison of all the models, as many as the uncorrelated subsets, and the selection of the ones that best explain the effect of transect variables on livestock consumption. Moreover, the AIC tool allowed us to conduct an explanatory analysis taking into account all possible predictor combinations. For each model, we calculated the Variance Inflation Factor (VIF) in order to detect collinearity among predictor variables (Zuur et al., 2010). To validate the final model, we tested for deviation from normality of the residual distribution by the Shapiro–Wilk normality test, for homoscedasticity by the Breush–Pagan test (Breusch and Pagan, 1979), and for residual autocorrelation by the Durbin–Watson test (Pires and Rodrigues, 2007).

3. Results

3.1. Genetic analysis

Genetic identifications of the 403 samples yield 205 (50.8%) reliable multilocus genotypes, corresponding to 58 wolves (31 males M and 27 females F), 5 dogs (4 M, 1 F), 9 wolf × dog hybrid individuals (8 M, 1 F). Wolf individuals were sampled from a minimum of 1 to a maximum of 10 times. The hybrids were sampled from 1 to 3 times while the 5 dogs once each.

Parentage analyses led to the assigning of 20–21 wolves to five distinct packs (Fig. 1, Table 1), respectively named: Imperia pack, Savona pack (on the border between Savona and Imperia), Beigua pack (in the Mount Beigua Regional Park, on the border between Savona and Genoa), Antola pack (in the Mount Antola Regional Park, in the province of Genoa) and Spezia pack. The minimum estimated territory sizes were

Table 1
Composition of the five packs detected in Liguria region by genetic analyses from 2008 to 2013.

Pack	Males	Females	Offspring of alpha pair	Adopted	Immigrants	Migration distance
Imperia	1	3	2	0	0	–
Savona	2–3	2	1–2	1	0	–
Beigua	3	2	1	2	2	97 and 75 km
Antola	3	2	1	2	1	122
Spezia	2	1	1	0	0	–

533 km^2 for Imperia pack, 779 km^2 for Savona pack, 144 km^2 for Antola pack, 83 km^2 for Beigua pack, and 101 km^2 for La Spezia pack. The remaining 37 wolves apparently were not related to any pack and were considered as floating or dispersing individuals (Caniglia et al., 2014). Thirteen dispersing wolves were resampled from one to nine times showing an average distance from the first to the last sampling of 19.9 km (SE = 5.19) with a maximum distance of 60.4 km.

3.2. Wolf diet

We analyzed a total of 1457 scats (year 1: 128; year 2: 276; year 3: 242; year 4: 350; year 5: 318; year 6:143) of which 863 were attributed to grazing season and 593 to the non-grazing one. Sample size was sufficient for each year and season according to the Brillouin index (minimum sample sizes: pooled years: 23 scats; year 1: 16; year 2: 19; year 3: 23; year 4: 19; year 5: 15; year 6: 15; grazing season: 17; non-grazing season: 19).

In view of the low proportion of scats attributed to dogs by genetic analyses (16 out of 389 fresh scats; 3.97%) we considered that errors did not influence the results. By scat analyses we detected 21 kinds of prey, pooled into eight categories (Table 2). Pooling the study years and the provinces, wild ungulates were the main food of wolves, followed by domestic ungulates. Other food categories showed a mean percent volume less than 3 for small mammals, medium sized mammals and grasses, and less than 1% for invertebrates, fruits, and garbage (Fig. 2). Among wild ungulates, the most consumed were wild boar and roe deer; the others species were less used (Fig. 3A). Among livestock species, wolves chiefly consumed goats, followed by cattle (mainly calves) and sheep; horse consumption was negligible (Fig. 3B).

3.3. Temporal variations of wolf diet

Two-way NPMANOVA showed significant changes in wolf diet according to years ($F = 13.31$; $P < 0.0001$) but not according to seasons ($F = 1.51$; $P = 0.153$), and a significant interaction between the two factors ($F = 43.56$; $P < 0.0001$). Significant differences resulted for all pairwise comparisons with exception of year 1 versus years 2, 3, 4, and 6, between years 2 and 3, and between years 5 and 6 (Table 2).

Livestock consumption increased significantly until year 3 and then decreased. Wild ungulates increased in the diet from the first to the last study year. Small mammals showed significant variation between years with a peak in the second one and the same was for the medium-sized mammals. Also for fruits, grasses, and garbage significant but moderate annual variations resulted (Table 2). The frequencies of occurrence of livestock and wild ungulates were negatively correlated (Spearman rank correlation: $r_s = -0.886$; $n = 6$; $P = 0.019$) and those of medium-sized and small mammals positively ($r_s = 0.941$; $n = 6$; $P = 0.005$).

Among livestock species, sheep consumption significantly decreased in the study period, while goat and cattle increased until the third year and then decreased (Table 2). Goats strongly contributed to the overall livestock consumption ($r_s = 0.943$; $n = 6$; $P = 0.005$). Concerning wild ungulate species, wild boar increased in the wolf diet until the fifth study year and then decreased, roe deer increased during the study

Table 2
Yearly variations of mean percent volume (MV%) and frequency of occurrence (FO%) of categories and prey species in wolf diet (Liguria region, N-Italy, 2008–2013).

Categories and species	Year 1		Year 2		Year 3		Year 4		Year 5		Year 6	
	n = 128		n = 276		n = 242		n = 350		n = 318		n = 143	
	MV%	FO%	MV%	FO%	MV%	FO%	MV%	FO%	MV%	FO%	MV%	FO%
Livestock	26.7	32.0	32.1	35.9	40.2	47.5	27.9	34.3	14.9	18.6	19.5	21.0
Ovisaries	5.5	19.5	4.4	13.1	4.6	15.7	3.1	12.5	2.3	13.6	0.1	3.3
Capra hircus	16.0	61.0	22.9	72.7	24.0	57.4	15.4	55.8	7.3	49.2	11.0	53.3
Bos taurus	5.3	19.5	4.4	13.1	11.7	27.0	9.1	30.8	5.3	37.3	8.4	43.3
Equus caballus	0.0	0.0	0.4	1.0	0.0	0.0	0.3	0.8	0.0	0.0	0.0	0.0
Wild ungulates	60.1	71.1	48.5	55.1	51.5	56.7	66.8	72.6	79.4	83.6	76.6	79.7
Sus scrofa	36.0	61.5	29.3	61.2	23.9	50.4	41.8	63.4	45.2	59.0	25.0	36.0
Capreolus capreolus	14.3	25.3	13.3	28.3	20.3	42.3	13.7	23.2	31.5	44.0	42.2	57.0
Cervus elaphus	4.0	7.7	1.8	3.3	4.5	8.0	5.2	7.9	0.2	0.4	0.6	0.9
Dama dama	5.9	11.0	3.8	12.5	2.9	5.1	4.4	7.5	1.2	1.5	8.1	11.4
Ovis aries musimons	0.0	0.0	0.4	0.7	0.0	0.0	0.3	0.4	0.0	0.0	0.0	0.0
Rupicapra rupicapra	0.0	0.0	0.0	0.0	0.0	0.0	1.4	2.0	1.4	1.9	0.7	0.9
Medium-sized mammals	4.4	7.0	8.0	12.7	4.4	5.4	4.0	0.9	0.6	0.9	0.7	1.4
Small mammals	2.1	2.3	5.9	7.6	1.9	5.0	0.4	0.9	0.5	0.9	1.4	1.4
Invertebrates	0.0	0.0	0.0	0.4	0.0	0.0	0.0	0.3	0.0	0.0	0.0	0.0
Fruits	0.1	0.8	1.5	7.6	0.8	1.7	0.9	1.1	0.9	1.6	0.0	0.0
Grasses	4.9	17.2	3.5	19.6	1.1	5.4	1.9	7.1	2.5	4.1	0.0	0.0
Garbage	0.0	0.0	0.0	0.0	0.0	0.0	0.1	1.1	0.0	0.0	0.0	0.0

NPMANOVA pairwise comparisons between years: 1–5 P = 0.003; 2–4 P = 0.002; 2–5 P = 0.002; 2–6 P = 0.002; 3–4 P = 0.005; 3–5 P = 0.002; 3–6 P = 0.002; 4–5 P = 0.005.
Livestock: H = 60.26; df = 5; P < 0.0001; wild ungulates: H = 108.55; df = 5; P < 0.0001; small mammals: H = 34.77; df = 5; P < 0.0001.
Medium-sized mammals: H = 68.66; df = 5, P < 0.0001; fruits: H = 40.07; df = 5, P < 0.0001; grasses: H = 77.07; df = 5; P < 0.0001.
Garbage: H = 12.67; df = 5; P = 0.027.
Ovis aries: H = 13.71; df = 5; P = 0.018; *Capra hircus*: H = 46.36; df = 5; P < 0.0001; *Bos taurus*: H = 14.63; df = 5; P < 0.012.
Sus scrofa: H = 49.56; df = 5; P < 0.0001; *Capreolus capreolus*: H = 90.20; df = 5; P < 0.0001; *Cervus elaphus*: H = 24.59; df = 5; P < 0.0001.
Dama dama: H = 20.95; df = 5; P = 0.001.

period, while red and fallow deer showed significant annual variations but without an evident trend (Table 2).

For livestock species we did not find significant seasonal changes, whereas among wild ungulate species significant differences resulted for wild boar (H = 34.37; df = 1; P < 0.0001) and for roe deer (H = 25.50; df = 1; P < 0.0001); in particular wild boar was more consumed in non-grazing season and, on the contrary, roe deer was more used in the grazing one (Table 3).

3.4. Variations in wolf diet between packs and dispersing wolves

Considering the five packs separately, we found overall significant differences in the use of food categories (NPMANOVA: F = 9.85; P = 0.0001); in particular the diet of Spezia pack was different from all the others. Moreover, we found significant differences comparing the diet of Beigua pack with those of Imperia and Antola. La Spezia pack consumed more livestock and medium-sized mammals and less wild ungulates and grasses than all the other packs (Table 4).

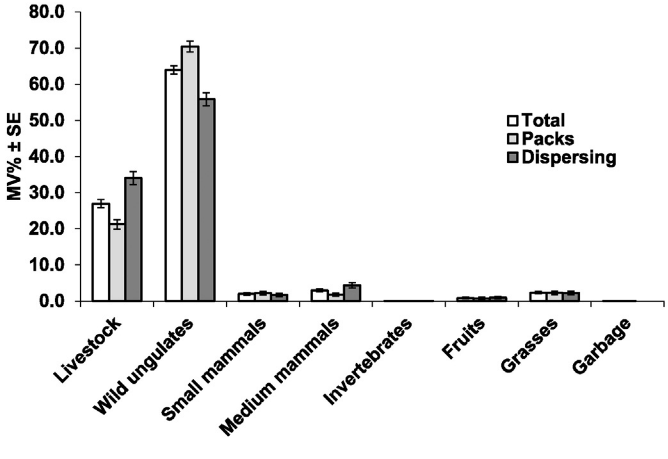

Fig. 2. Mean percent volume ± SE of prey categories in wolf diet in Liguria from 2008 to 2013.

Among livestock species, we detected significant differences for goats and cattle, the first species being more used by Spezia pack, and the second by Imperia and Spezia ones. Also the use of wild ungulate

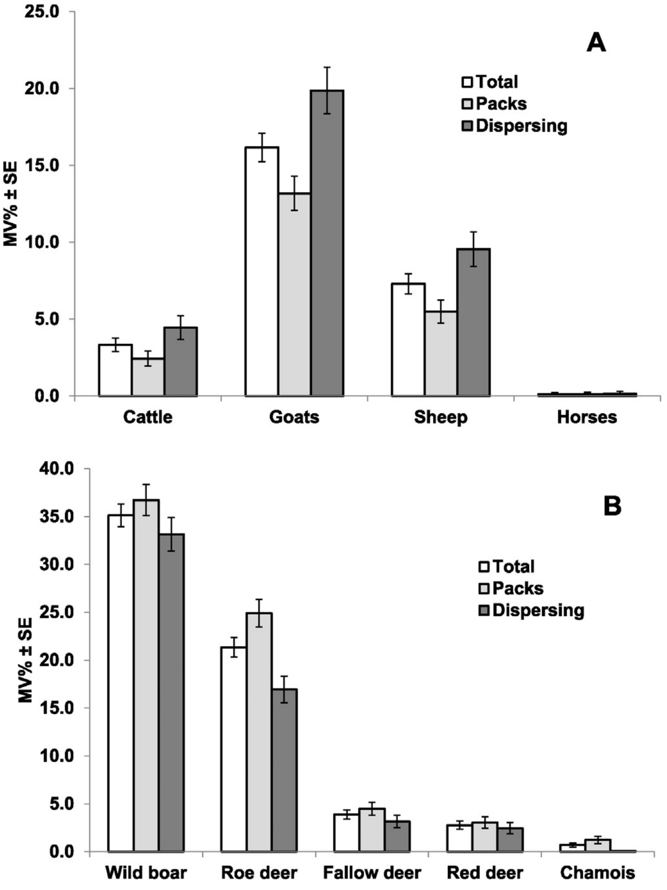

Fig. 3. Mean percent volume ± SE of livestock (A) and wild ungulate species (B) in wolf diet (Liguria region 2008–2013).

Table 3
Seasonal variations of mean percent volume (MV%) and frequency of occurrence (FO%) of categories and prey species in wolf diet (Liguria region, N-Italy, 2008–2013).

Categories and species	Grazing season		Non-grazing season	
	n = 863		n = 593	
	MV%	FO%	MV%	FO%
Livestock	28.1	32.0	25.1	29.2
Ovisaries	2.9	11.2	3.9	18.5
Capra hircus	17.1	62.7	14.8	59.0
Bos taurus	8.1	28.6	6.2	25.4
Equus caballus	0.1	0.4	0.2	0.6
Wild ungulates	62.7	68.7	65.9	71.0
Sus scrofa	29.2	48.9	43.7	68.2
Capreolus capreolus	25.5	43.5	15.6	25.4
Cervus elaphus	3.3	5.4	2.0	3.1
Dama dama	4.2	7.8	3.4	6.2
Ovis aries musimons	0.0	0.0	0.3	0.5
Rupicapra rupicapra	0.5	0.8	1.0	1.4
Medium-sized mammals	3.1	4.8	2.7	4.0
Small mammals	2.2	3.4	1.6	2.5
Invertebrates	0.0	0.2	0.0	0.0
Fruits	0.6	1.9	1.1	3.2
Grasses	1.9	7.1	2.9	11.1
Garbage	0.02	0.2	0.01	0.3

species resulted different between packs; in particular wild boar were consumed mainly by Imperia and Beigua packs, roe deer by Savona and Beigua packs, red deer by Savona pack, fallow deer by Savona and Antola packs, and chamois by Imperia pack (Table 4).

Comparing the diets of individuals belonging to a pack and the dispersing ones, we found overall significant differences in the use of prey categories (NPMANOVA: F = 32.24; P < 0.0001). In particular, a higher consumption of livestock (H = 29.44; df = 1; P < 0.0001) and medium-sized mammals (H = 10.98; df = 1; P = 0.001) resulted for dispersing wolves, whereas the contrary was the case for wild ungulate use, higher in pack diet than in that of dispersing individuals (H = 40.01; df = 1; P < 0.0001) (Fig. 2). Significant differences resulted also considering livestock and wild ungulate species. In particular goats and cattle were more consumed by dispersing individuals (H = 9.17; df = 1; P = 0.002 and H = 7.65; df = 1; P = 0.006 respectively) but packs consumed more roe deer (H = 15.33; df = 1; P = 0.0001) and chamois (H = 5.67; df = 1; P = 0.017) (Fig. 3A and B). The diet of dispersing wolves differed significantly from that of each pack (P ≤ 0.003 for all pairwise comparisons), with the exception of Spezia pack (P = 0.623).

3.5. Livestock use versus availability

The livestock density was greater in pack territories than in the remaining part of the study area with the exception of Antola and Savona packs (Table 5). During the study period we recorded a total of 176 predations on livestock 15 of which upon cattle and 161 upon sheep and goats. Considering predation cases on livestock species we found significant differences between observed and expected frequencies of events across packs and dispersing wolves for cattle (χ^2 = 4167.78; df = 5; P < 0.0001), sheep and goats (χ^2 = 4107.74; df = 5; P < 0.0001), and for the species pooled (χ^2 = 187.83; df = 5; P < 0.0001). In particular cattle was preyed in proportion to the availability by Antola and Savona packs, avoided by Beigua, Imperia and Spezia packs, and used more than the availability by dispersing wolves. Sheep and goats were used as available by Antola and Spezia packs, underused by Beigua, Imperia, and Savona packs, and overused by dispersing wolves. Pooled species were used in proportion to the availability by Antola pack, less than the availability by the other packs, and more than the availability by dispersing wolves (Table 6). Considering the packs pooled the frequency of predation events was significantly less than expected for sheep and goats (χ^2 = 97.73; df = 1; P < 0.0001), and for the species pooled (χ^2 = 103.64; df = 1; P < 0.0001) but for cattle (χ^2 = 4.29; df = 1; P = 0.066); pooled packs underused cattle, sheep and goats, and the pooled species (Table 6).

3.6. Model of livestock consumption

By the Multiple Linear Regression Analyses on the subsets of uncorrelated predictors, we obtained only one model, the others having

Table 4
Mean percent volume (MV%) and frequency of occurrence (FO%) of categories and prey species in the diet of wolf packs (Liguria region, N-Italy, 2008–2013).

Categories and species	Imperia pack		Savona pack		Beigua pack		Antola pack		La Spezia pack	
	n = 297		n = 102		n = 213		n = 137		n = 64	
	MV%	FO%	MV%	FO%	MV%	FO%	MV%	FO%	MV%	FO%
Livestock	24.2	27.9	17.6	20.6	13.1	14.6	18.2	27.0	46.0	50.0
Ovis aries	2.6	14.5	1.9	9.5	2.9	22.6	1.7	16.2	2.9	6.3
Capra hircus	11.7	53.0	13.1	76.2	8.2	64.5	12.9	70.3	37.2	81.3
Bos taurus	9.7	42.2	2.6	14.3	2.0	19.4	3.6	16.2	5.9	12.5
Equus caballus	0.3	1.2	0.0	0.0	0.0	0.0	0.0	0.0	0.0	0.0
Wild ungulates	69.7	73.1	75.0	83.3	81.3	84.5	64.6	78.1	44.4	50.0
Sus scrofa	45.3	66.4	19.2	27.1	38.7	51.7	30.0	47.7	31.6	75.0
Capreolus capreolus	19.8	29.5	35.1	48.2	35.2	48.9	19.3	33.6	11.3	25.0
Cervus elaphus	1.6	2.3	9.8	12.9	3.6	4.4	1.9	2.8	0.0	0.0
Dama dama	0.0	0.0	10.0	11.8	3.8	5.6	13.4	24.3	0.0	0.0
Ovis aries musimons	0.3	0.5	0.0	0.0	0.0	0.0	0.0	0.0	0.0	0.0
Rupicapra rupicapra	2.6	3.7	1.0	1.2	0.0	0.0	0.0	0.0	1.5	0.0
Medium-sized mammals	1.0	1.0	0.4	1.0	1.0	1.4	3.1	8.0	7.7	7.8
Small mammals	2.2	3.0	3.3	0.0	0.9	0.9	4.3	5.1	0.0	7.8
Invertebrates	0.002	0.3	0.0	0.0	0.0	0.0	0.0	0.0	0.0	0.0
Fruits	0.1	0.3	1.4	3.9	0.0	0.0	2.9	8.0	0.2	1.6
Grasses	0.4	5.4	2.4	4.9	1.9	7.0	7.9	19.7	0.3	4.7
Garbage	0.02	0.7	0.2	2.0	0.0	0.0	0.0	0.0	0.0	0.0

NPMANOVA pairwise comparisons between packs: Imperia–Beigua P = 0.008; Imperia–La Spezia P = 0.001; Savona La Spezia P = 0.001; Beigua–Antola P = 0.01; Beigua–La Spezia P = 0.001; Antola–La Spezia P = 0.001.
Livestock: H = 36.31; df = 4; P < 0.0001; wild ungulates: H = 45.59; df = 4; P < 0.0001; medium-sized mammals: H = 24.97; df = 4; P < 0.0001.
Fruits: H = 34.10; df = 4; P < 0.0001; grasses: H = 31.59; df = 4; P < 0.0001.
Capra hircus: H = 38.08; df = 4; P < 0.0001; *Bos taurus*: H = 20.54; df = 4; P < 0.0001.
Sus scrofa: H = 27.26; df = 4; P < 0.0001; *Capreolus capreolus*: H = 34.92; df = 4; P < 0.0001; *Cervus elaphus*: H = 24.17; df = 4; P < 0.0001.
Dama dama: H = 69.57; df = 4; P < 0.0001; *Rupicapra rupicapra*: H = 9.53; df = 4; P = 0.049.

Table 5
Percentage of pastures and density (heads per km^2) of livestock species in pack territories and in non-pack area.

Pack	Pastures (%)	Cattle	Sheep and goats	Total
Antola	7.6	1.4	0.1	1.5
Beigua	4.7	1.9	3.3	5.2
Imperia	8.5	3.7	14.1	17.8
Savona	1.8	0.5	0.5	1.0
Spezia	8.6	7. 5	2.5	10.0
Pooled packs	4.7	2.1	5.1	7.3
No pack	2.6	0.8	1.7	2.5

Table 7
Results of multiple regression analysis of mean percent volume of domestic ungulates in the wolf diet vs. the transect variables (N = 34).

Transect variables	Regression coefficients (SE)	Standardized coefficients	t	P	VIF
Intercept	57.2 (6.16)		9.29	<0.0001	
Pack presence	−31.4 (3.28)	−0.85	9.59	<0.0001	1.3
Pasture number	0.9 (0.15)	0.56	6.45	<0.0001	1.2
Prevention (%)	−37.5 (7.08)	−0.45	5.29	<0.0001	1.1
Roe deer abundance (IKA)	−15.7 (3.92)	−0.36	3.99	0.001	1.3
Deciduous woods (%)	−0.4 (0.12)	−0.29	3.48	0.002	1.1
Wild Ungulate Diversity Index	9.8 (4.27)	0.21	2.29	0.031	1.4

$R^2 = 0.807$. SEE = 7.68. F = 22.59. df = 6,25. $P < 0.0001$.

ΔAICc > 2 (Table 7). Six variables with significant regression coefficients entered the model explaining 80.7% of the variance of the mean percent volume of livestock in the wolf diet. The presence of packs, prevention methods, deciduous woods and roe deer abundance had a negative effect on livestock consumption, whereas the number of pastures in the areas covered by transects and the diversity of wild ungulate community had a positive effect (Fig. 4). The presence of a pack (by opposition to dispersing wolves) had the strongest influence followed by the number of pastures, the percentage of pastures with prevention methods, roe deer abundance, percentage of deciduous forest, and finally wild ungulate diversity index (Table 7). The Variance Inflation Factor revealed no collinearity among predictors, and the residuals of the model were normally distributed (Shapiro–Wilk test = 0.98, P = 0.736), not auto-correlated (Durbin–Watson statistic = 2.30) and the homoscedasticity was respected (Breush–Pagan statistic = 6.32; df = 6; P = 0.389). Examining the relationships between predictors, transects with wolf packs were characterized by a greater presence of pastures, percentage of deciduous woods, and roe deer abundance in respect to those with dispersing wolves, whereas the percentage of livestock farms with prevention methods and the wild ungulate diversity index were lower (Fig. 5). However these differences were not significant (Mann–Whitney U test, P > 0.05 in all cases).

4. Discussion

The diet of wolves in the Liguria region is characterized by a medium–high occurrence of wild ungulates and by an important part consisting of large domestic prey, the other food categories being a negligible fraction of the diet. This picture places the food habits of wolves in our study area between those of populations preying almost exclusively on wild herbivores and those of wolves living mainly at the expense of livestock and other food of human origin, that can be found in human altered landscapes of some South and East European countries such as Portugal, Spain, South-central Italy, Greece, Bulgaria, Ukraine, Moldova, and Azerbaijzan (Meriggi and Lovari, 1996; Meriggi et al., 2011; Migli et al., 2005; Papageorgiou et al., 1994; Peterson and Ciucci, 2003; Vos, 2000; Zlatanova et al., 2014). Usually the former are found in areas where there are rich and abundant wild ungulate guilds and where livestock is inaccessible because of the husbandry methods, and the latter where wild ungulates are rare and livestock is free ranging and unguarded (Cuesta et al., 1991; Meriggi and Lovari, 1996; Okarma, 1995; Peterson and Ciucci, 2003; Zlatanova et al., 2014). Considering Europe as a whole, the importance of wild ungulates in the wolf diet seems to follow a cline decreasing from North to South and an increasing trend in particular after the eighties in the last century (Meriggi and Lovari, 1996; Meriggi et al., 2011; Okarma, 1995; Zlatanova et al., 2014). Wolves in the Liguria region use fewer wild ungulates and more livestock than those of other close areas located in the northern Apennines (Capitani et al., 2004; Mattioli et al., 1995, 2004, 2011; Meriggi et al., 1996, 2011, 2015; Milanesi et al., 2012). These differences can be related to the characteristics of wild prey community in Liguria where there are two widespread species locally very abundant (wild boar and roe deer), other two localized but with high density populations (fallow deer and chamois) and the last two (red deer and mouflon) are at present rare. Moreover few livestock farms adopt prevention methods, leaving herds, in particular goat flocks, free ranging and unguarded on pastures during the grazing season.

This situation could also cause the annual variations of the use of wild ungulates and livestock; both being probably related to the fluctuations in abundance of the main wild prey species (wild boar and roe deer) because of the quite constant number of livestock heads reared in the Liguria region. The close negative relationships between the importance of the two main food categories in the wolf diet over the study period demonstrates that livestock is more used when wild herbivores are less available (Meriggi and Lovari, 1996; Meriggi et al., 2011). In any case, wild herbivores showed an increase in the wolf diet during the study period in accordance with the general trend already found in Europe and in particular in Italy, and in agreement with the ability of wolves to respond in a short time to the changes in abundance of the preferred prey species (Meriggi and Lovari, 1996; Meriggi et al., 2011, 2015; Peterson and Ciucci, 2003).

Wolves in Liguria consumed mainly wild boar, the main wild prey in the Mediterranean range of the wolf (Meriggi and Lovari, 1996; Meriggi et al., 2011; Okarma, 1995; Zlatanova et al., 2014). This choice could be due to the high wild boar abundance, and to the fact that the species lives in large groups easily detectable by a predator. Furthermore, births occur all year round, causing the removal from the matriarchal groups of sub-adults; these individuals are profitable prey because they have the body size of an adult without its experience so that the handling time can be minimized (Meriggi et al., 1996, 2011, 2015; Milanesi et al., 2012).

The second wild ungulate in order of importance was the roe deer; roe deer has solitary behavior so its detectability and its encounter rate are low compared to the wild boar, with the exception of the areas where the species is present with high density (Meriggi et al.,

Table 6
Results of Bonferroni simultaneous confidence interval analyses for the differences between expected (EUP) and observed usage proportion (OUP) of livestock species across packs and dispersing wolves.

Pack	Cattle (n = 15)		Sheep and goats (n = 161)		Pooled species (n = 176)	
	EUP	OUP	EUP	OUP	EUP	OUP
Antola	0.030	0.133	0.001	0.012	0.010	0.023
Beigua	0.024	0.000[a]	0.019	0.000[a]	0.020	0.000[a]
Imperia	0.302	0.000[a]	0.511	0.019[a]	0.447	0.017[a]
Savona	0.060	0.067	0.026	0.000[a]	0.037	0.006[a]
Spezia	0.116	0.000[a]	0.017	0.012	0.048	0.011[a]
Pooled packs	0.532	0.200[a]	0.575	0.043[a]	0.562	0.057[a]
No pack	0.468	0.800[a]	0.425	0.957[a]	0.438	0.943[a]

[a] Differences at minimum level of $\alpha = 0.05$.

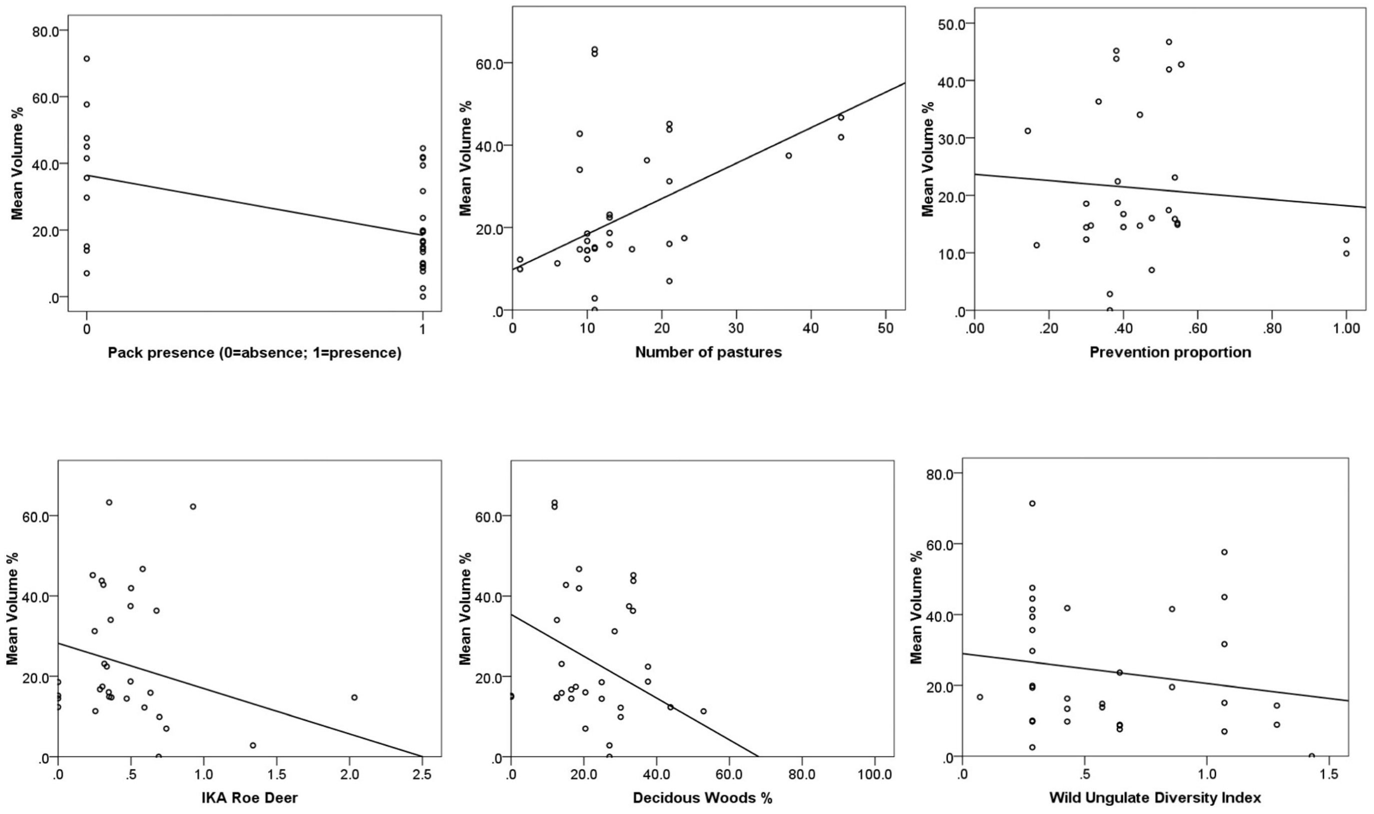

Fig. 4. Relationships between mean volume (%) of livestock in wolf diet and the transect variables entered in the regression model.

2011, 2015; Milanesi et al., 2012). Among the other wild ungulate species only fallow deer reached a limited importance in the last study year; this species, together with the chamois, is locally abundant but the latter is more difficult to prey upon because of the low accessibility of the habitats (Meriggi and Lovari, 1996; Patalano and Lovari, 1993; Poulle et al., 1997). Red deer and mouflon are used only occasionally

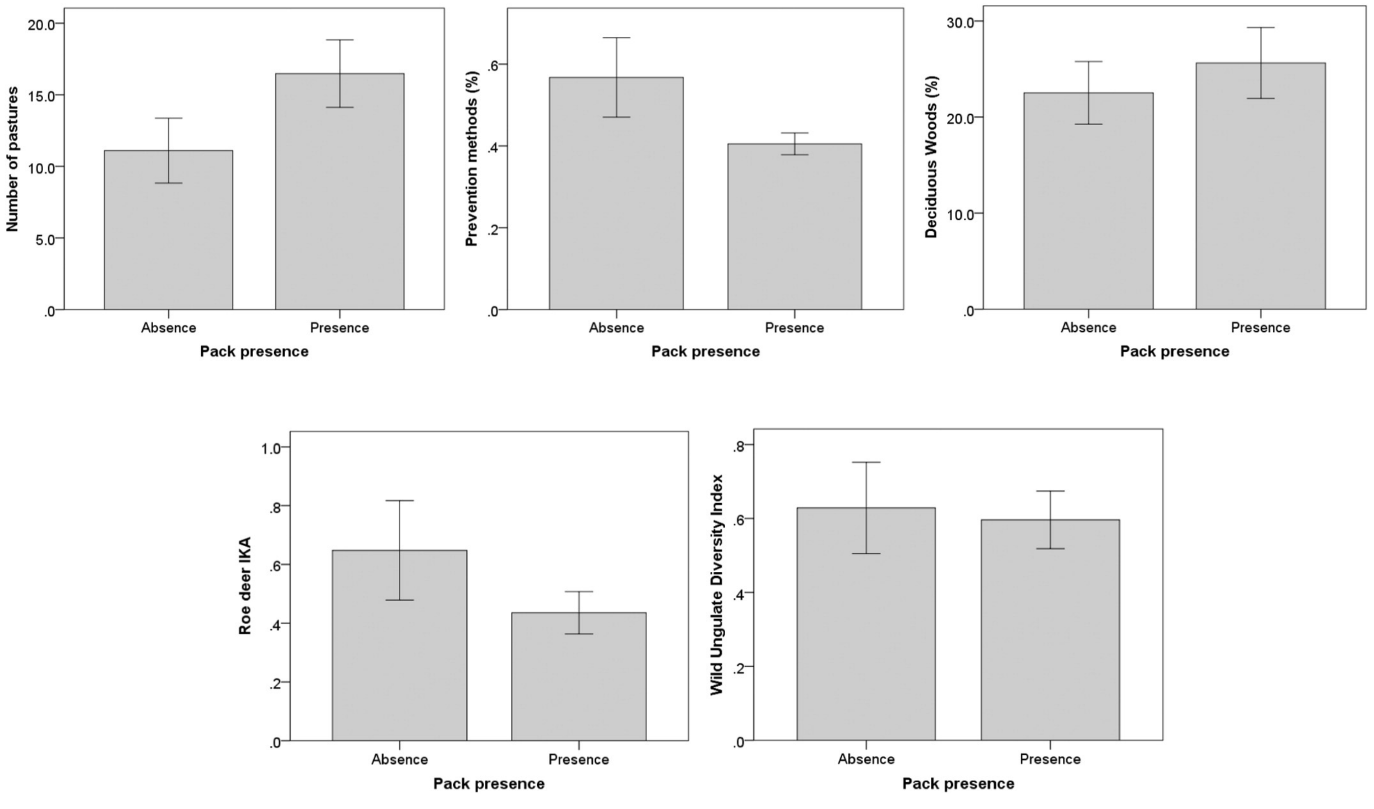

Fig. 5. Average values (±SE) of transect variables entered the model of livestock consumption in relation to the pack presence.

because of their rarity. Despite the annual variations of the wild ungulate species in the wolf diet, only for roe deer did we find a trend with an increase of three times the importance from the first to the last study year. This is in accordance with the general increase of the Italian population of roe deer in the last decades (Carnevali et al., 2009).

The importance of livestock species in the wolf diet in Liguria region was not in agreement with their respective abundance; indeed, the main prey species were goats and cattle that are respectively the third and the second in number. Goats are particularly vulnerable to wolf predation because they are left unguarded and free ranging on mountains; moreover goats lost in the mountain can survive, forming groups of feral animals, available all year round for wolves, and these groups of feral goats are increasing in number and size in Liguria. As far as cattle are concerned, wolves prey almost exclusively upon calves born during the grazing period on pastures, whereas adult cows are rarely attacked; so only cattle farms that adopt calf births on pastures are vulnerable (Brangi et al., 1992; Meriggi et al., 1991, 1996).

Surprisingly, we did not find significant changes of the food categories from grazing to non-grazing seasons, with the exception of fruits that were more eaten in the grazing one. As for wild ungulates, wolves used wild boar in winter and roe deer in summer; in summer, roe deer are more vulnerable because of the presence of young, and in winter, several wild boars are injured and not retrieved by hunters during drive hunts and consequently are easily found and caught by wolves. Moreover snow depth makes wild boars more vulnerable to predation (Okarma, 1995). As for livestock, we found that wolves eat it, particularly goats, also in winter, although in this season they should be in the sheepfolds. This pattern was also found by Patalano and Lovari (1993) in the Abruzzo National Park (central Italy). Two reasons can be proposed: firstly, the scat analysis does not permit the making of distinctions between consumption of preyed animals and of carcasses, thus wolves can feed on carcasses of lost animals during winter that have been well conserved by snow; also shepherds sometime leave their livestock on pastures for a part of winter, exposing it to wolf attacks during the cold season.

The highlighted differences of the diets between packs seem to be partially related to the local variation of wild ungulate species, and consequently to the composition of the wild ungulate community. In particular, the packs located in the provinces with the highest density of roe deer (Savona and the western part of Genoa, respectively 38.6 and 46.7 individuals per km^2) consumed more roe deer than the other packs; moreover, fallow deer and chamois occurred almost exclusively in the diet of packs living in the areas where these species are present or abundant. Moreover, Spezia pack has a diet with a high percentage of livestock compared to the other packs. In this case, pack adaptation to feeding on livestock could be the result of the scarcity of wild prey (Meriggi and Lovari, 1996; Meriggi et al., 2011; Vos, 2000). However packs do not hunt only according to prey abundance, but accessibility, vulnerability and profitability of prey as well as composition of ungulate community, wolf foraging behavior, previous hunting experience, cultural transmission, and learning from parents can heavily affect food choice and predator diet (Curio, 1976; Endler, 1991; Huggard, 1993; Meriggi et al., 1996).

We also found that the packs consume more wild ungulates than the dispersing wolves, and dispersing individuals showed a greater use of livestock than packs. Dispersing wolves are mainly young individuals and their hunting success is usually lower than that of older ones; because of this they could direct predation on livestock that, because of domestication, have less effective defenses against predators than wild large prey (Meriggi and Lovari, 1996; Meriggi et al., 1996). Moreover, dispersing wolves can cover great distances in a short time and therefore do not have the time to learn the wild prey distribution (Linnell et al., 1999); as a consequence dispersing individuals can attack livestock herds that have a greater detectability because of their highly clumped distribution and their small movement. This finding is confirmed by the use vs. availability analyses that showed selection for livestock species by dispersing wolves whereas packs underuse or use as availability both cattle or sheep and goats or the species pooled.

The model of livestock consumption explained a high percentage of the variance and it was therefore very informative. The presence of packs, unlike the case of dispersing individuals, had a negative effect on livestock consumption. This is in accord with the lower use of livestock species that we found in the pack diet compared to that of dispersing wolves; structured packs hunt on their territory and know where to find wild prey, whereas dispersing individuals, new to the area, do not know it and hunt the first potential prey they encounter. The number of pastures had a positive effect on consumption of livestock because if the pastures are fragmented and scattered in the forests, the contact zone between woods and pastures increases and this can enhance the predation risk facilitating the attacks by wolves (Dondina et al., 2014; Kaartinen et al., 2009; van Lière et al., 2013). Prevention methods negatively affected the livestock consumption; the effect of the adoption of different methods (nocturnal shelter, presence of shepherds and dogs, electric fences) of herd and flock protection in reducing predator attacks and their success was demonstrated by several studies even if in some cases they fail or are impossible to adopt (Dondina et al., 2014; Espuno et al., 2004; Landry et al., 1999; Mech and Boitani, 2003; Miller, 2015). Roe deer abundance also decreased the use of livestock; the presence of roe deer corresponds to the presence of a second potential prey species for wolves, the first one being wild boar that is present in the whole region at high density. In this situation, if the abundance of one of the two species drops, wolves can compensate with the other one to satisfy their food requirements and thus neglect livestock. In particular, wild boar populations are subject to substantial fluctuations related to the occurrence of mast seeding years and the presence of a secondary prey at medium–high density can stabilize the overall availability of prey species (Bieber and Ruf, 2005). This is in accord with Meriggi and Lovari (1996) and with Meriggi et al. (2011) which suggest the increase of the diversity of wild ungulate community as a measure to mitigate the conflicts with husbandry. The extent of deciduous woods decreased livestock consumption, probably in relation to the great density of wild ungulates (wild boar and deer) that can be find in this kind of vegetation (Focardi et al., 2009; Fonseca, 2008); in fact, usually the presence of large wild herbivores decreases predation on livestock by wolves (Meriggi and Lovari, 1996; Meriggi et al., 1996, 2011). Surprisingly, the diversity of wild ungulate community had a positive effect on livestock use, a rich and abundant community of wild ungulates reducing the consumption of livestock elsewhere (Meriggi et al., 1996; Meriggi and Lovari, 1996). In our case the wild ungulate diversity index was positively related to the presence of chamois; this species is very localized and abundant only in the Imperia province where the roe deer density and wild boar abundance are lower than in other parts of Ligurian region. If wolves have the choice between chamois and easier prey, e.g. livestock, they will prefer the latter.

5. Conclusion

In Liguria, as in many countries of southern Europe, conflicts between wolf conservation and husbandry are far from being solved and they are an important threat to wolf conservation, as the high number of wolves found illegally killed demonstrates (12 individuals out of 16 confirmed dead between 2007 and 2014 in the whole region). Poaching by shooting and poisoning is the main mortality factor of wolves in the region and in Italy, and can be related to the damage to livestock farms (Lovari et al., 2007). Consequently it is important for wolf conservation to adopt management options that can effectively protect Ligurian wolves, to maintain a connection between sub-populations of Alps and Apennines, avoiding the isolation of Alpine wolves, and to permit the linkup between Italian and Balkan populations.

The main results of our study useful to improving wolf conservation and planning effective management actions aimed at conflict mitigation are as follows: i) the relationship between livestock and wild ungulate consumption, ii) the differences in livestock and wild ungulate use between packs, iii) the differences in diet between packs and dispersing wolves, and iv) the model of livestock consumption showing that the main factors negatively affecting predation upon livestock species are the presence of packs, the adoption of prevention methods, and roe deer abundance.

In order to limit the damage that wolves cause on husbandry, conservation measures should be primarily aimed at restoring a rich and abundant wild ungulate community. This goal can be achieved by a better regulation of wild boar and roe deer hunting and by more effective harvest plans in order to maintain stable the population of the former, and to increase the density of the latter, in particular in those areas where it is at low density. Moreover, reintroductions of red deer should be carried out to increase its presence in the region.

Another important step for conflict mitigation is to encourage the presence of wolf packs that at present are limited in number in respect to the availability of suitable areas in the region (Meriggi et al., 2013, unpublished report). If all available and suitable areas were occupied by packs the presence of erratic wolves would be reduced because of the intolerance of packs members towards foreign individuals (Mech, 1970; Mech and Boitani, 2003), and livestock depredation lowered. To enhance the pack numbers in Liguria the main action is poaching repression; illegal kills can cause pack break up and social disruption with an increase of dispersal and the formation of new breeding pairs in other areas, the ultimate effect of this being a low effectiveness of wild prey use and a consequent increase of livestock depredation (Haber, 1996; Sand et al., 2006; Wielgus and Peebles, 2014).

Prevention methods are important in reducing livestock consumption but they are not applicable everywhere, in particular on very large pastures and because of the increased costs of breeding.

On the basis of our results numeric control seems to be questionable. In a pack, removal of one of the two alpha members can lead to its break up and the other individuals leave the territory (Mech and Boitani, 2003). Consequently, livestock attacks can decrease drastically, the wolves not being any longer present in the area. However, it is a brief effect because empty suitable areas are rapidly recolonized by dispersing individuals, who have a bigger consumption of livestock than packs; these dispersing individuals should quickly change into residents and form a pack but this process requires several years during which livestock depredation increases. So removal measures do not solve the problem in the long run but conversely they can amplify it also putting at risk the wolf population because of the direct and indirect effects of harvest on recruitment (Ausband et al., 2015; Wielgus and Peebles, 2014). Use of prevention tools, promotion of a rich wild ungulate community and avoiding numerical control and poaching must be used together to mitigate conflicts between wolf conservation and husbandry. They have to be combined with good monitoring of wolf populations, so that which stage of colonization wolves are at can be known.

Acknowledgments

This research was supported by the Liguria Region, by the Regional Park "M. Antola" (Regional Operational Program — European Regional Development Fund 2007–2013 - CUP B37E12000040009). We thank F. Puopolo, L. Schenone, D. Signorelli, and M. Zanzottera for their help in field work as well as the personnel of the Forestry Corp and Provincial Police who helped in collecting data. V. Devictor, S. Lovari, and an anonymous referee provided helpful comments to an early draft of the manuscript.

Appendix A. Environmental variables measured in the 13-km buffers around the transects and used to model livestock consumption

Environmental variables	Unit
Mixed woods	%
Deciduous woods	%
Coniferous woods	%
Scrublands	%
Pastures	%
Rocky areas	%
Water (streams and lakes)	%
Urban areas	%
Altitude	Meters a.s.l.
Slope	Degree
Annual insolation	kW/m^2

References

Akaike, H., 1973. Maximum likelihood identification of Gaussian autoregressive moving average models. Biometrika 60, 255–265.

Anderson, M.J., 2000. NPMANOVA: A FORTRAN Computer Program for Non-parametric Multivariate Analysis of Variance (for any two-factor ANOVA design) Using Permutation Tests. Department of Statistics, University of Auckland, Auckland, New Zealand.

Anderson, M.J., 2001. A new method for non-parametric multivariate analysis of variance. Austral Ecol. 26, 32–46.

Anderson, D.R., Burnham, K.P., Thompson, W.L., 2000. Null hypothesis testing: problems, prevalence and an alternative. J. Wildl. Manag. 64, 912–923.

Anderson, D.R., Link, W.A., Johnson, D.H., Burnham, K.P., 2001. Suggestion for presenting the results of data analyses.J. Wildl. Manag. 65, 373–378.

Ansorge, H., Kluth, G., Hahne, S., 2006. Feeding ecology of wolves *Canis lupus* returning to Germany. Acta Theriol. 51, 99–106.

Apollonio, M., Mattioli, L., Scandura, M., Mauri, L., Gazzola, A., Avanzinelli, E., 2004. Wolves in the Casentinesi forests: insights for wolf conservation in Italy from a protected area with a rich wild prey community. Biol. Conserv. 120, 249–260.

Ausband, D.E., Stansbury, C.R., Stenglein, J.L., Struthers, J.L., Waits, L.P., 2015. Recruitment in a social carnivore before and after harvest. Anim. Conserv. 18, 415–423.

Balciauskas, L., 2008. Wolf numbers and distribution in Lithuania and problems of species conservation. Ann. Zool. Fenn. 45, 329–334.

Barabesi, L., Fattorini, L., 2013. Random versus stratified location of transects or points in distance sampling: theoretical results and practical considerations. Environ. Ecol. Stat. 20, 215–236.

Barabesi, L., Franceschi, S., 2011. Sampling properties of spatial total estimators under tessellation stratified designs. Environmetrics 22, 271–278.

Behdarvand, N., Kaboli, M., Ahmadi, M., Nourani, E., Mahini, A.S., Aghbolaghi, M.A., 2014. Spatial risk model and mitigation implications for wolf–human conflict in a highly modified agroecosystem in western Iran. Biol. Conserv. 177, 156–164.

Best, D.J., Rayner, J.C., 2007. Chi-squared components for tests of fit and improved models for the grouped exponential distribution. Comput. Stat. Data Anal. 51, 3946–3954.

Bieber, C., Ruf, T., 2005. Population dynamics in wild boar *Sus scrofa*: ecology, elasticity of growth rate and implications for the management of pulsed resource consumers. J. Appl. Ecol. 42, 1203–1213.

Boitani, L., 2000. Action plan for the conservation of wolves in Europe (*Canis lupus*). Nature and Environment, No. 113. Council of Europe Publishing.

Brangi, A., Rosa, P., Meriggi, A., 1992. Predation by wolves (*Canis lupus*) on wild and domestic ungulates in northern Italy. In: Spitz, F., Janeau, G., Gonzales, G., Alaignier, S. (Eds.), International Symposium "Ongulés/Ungulates 91". Toulouse, pp. 541–543.

Breitenmoser, U., 1998. Large predators in the Alps: the fall and rise of man's competitors. Biol. Conserv. 83, 279–289.

Breusch, T.S., Pagan, A.R., 1979. A simple test for heteroscedasticity and random coefficient variation. Econometrica 47, 1287–1294.

Brillouin, L., 1956. Science and Information Theory. Academic Press, NewYork, USA.

Brunner, H., Coman, B.J., 1974. The Identification of Mammalian Hair. Inkata Press, Melbourne, Australia.

Burnham, K.P., Anderson, D.R., 2002. Model Selection and Multimodel Inference: A Practical Information-Theoretic Approach. second ed. Springer-Verlag, New York, USA.

Calenge, C., 2006. The package "adehabitat" for the R software: a tool for the analysis of space and habitat use by animals. Ecol. Model. 197, 516–519.

Caniglia, R., Fabbri, E., Cubaynes, S., Gimenez, O., Lebreton, J.D., Randi, E., 2012. An improved procedure to estimate wolf abundance using non-invasive genetic sampling and capture–recapture mixture models. Conserv. Genet. 13, 53–64.

Caniglia, R., Fabbri, E., Mastrogiuseppe, L., Randi, E., 2013. Who is who? Identification of livestock predators using forensic genetic approaches. Genetics 7, 397–404.

Caniglia, R., Fabbri, E., Galaverni, M., Milanesi, P., Randi, E., 2014. Noninvasive sampling and genetic variability, pack structure, and dynamics in an expanding wolf population. J. Mammal. 95, 41–59.

Capitani, C., Bertelli, I., Varuzza, P., Scandura, M., Apollonio, M., 2004. A comparative analysis of wolf (*Canis lupus*) diet in three different Italian ecosystems. Mamm. Biol. 69, 1–10.

Carnevali, L., Pedrotti, L., Riga, F., Toso, S., 2009. Banca dati Ungulati. Status, distribuzione,consistenza, gestione e prelievo venatorio delle popolazioni di Ungulati in Italia. Rapporto 2001–2005. Biol. Conserv. Fauna 117, 1–168.

Chapron, G., Legendre, S., Ferrière, R., Clobert, J., Haight, R.G., 2003. Conservation and control strategies for the wolf (*Canis lupus*) in Western Europe based on demographic models. C. R. Biol. 326, 575–587.
Chapron, G., Kaczensky, P., Linnell, J.D., Von Arx, M., Huber, D., Andrén, H., ... Boitani, L., 2014. Recovery of large carnivores in Europe's modern human-dominated landscapes. Science 346, 1517–1519.
Ciucci, P., Boitani, L., Francisci, F., Andreoli, G., 1997. Home range, activity and movements of a wolf pack in central Italy. J. Zool. 243, 803–819.
Corsi, F., Duprè, E., Boitani, L., 1999. A large-scale model of wolf distribution in Italy for conservation planning. Conserv. Biol. 13, 150–159.
Cuesta, L., Barcena, F., Palacios, F., Reig, S., 1991. The trophic ecology of the Iberian Wolf (*Canis lupus signatus* Cabrera,1907) — a new analysis of stomach's data. Mammalia 55, 239–255.
Curio, E., 1976. The Ethology of Predation. Springer, Berlin, Germany.
De Marinis, A.M., Asprea, A., 2006. Hair identification key of wild and domestic ungulates from southern Europe. Wildl. Biol. 12, 305–320.
Debrot, S., Mermod, C., Fivaz, G., Weber, J.M., 1982. Atlas des poils de mammifères d'Europe. Insitut de Zoologie de l'Université de Neuchâtel, Neuchâtel, Swiss.
Dondina, O., Meriggi, A., Dagradi, V., Perversi, M., Milanesi, P., 2014. Wolf predation on livestock in an area of northern Italy and prediction of damage risk. Ethol. Ecol. Evol. 27, 200–219.
Eggermann, J., da Costa, G.F., Guerra, A.M., Kirchner, W.H., Petrucci-Fonseca, F., 2011. Presence of Iberian wolf (*Canis lupus signatus*) in relation to land cover, livestock and human influence in Portugal. Mamm. Biol. 76, 217–221.
Endler, J.A., 1991. Interactions between predators and prey. In: Krebs, J.R., Davies, N.B. (Eds.), Behavioural Ecology. An Evolutionary Approach. Blackwell Sci. Publ., Oxford, pp. 169–196.
Espuno, N., Lequette, B., Poulle, M.L., Migot, P., Lebreton, J.D., 2004. Heterogeneous response to preventive sheep husbandry during wolf recolonization of the French Alps. Wildl. Soc. Bull. 32, 1195–1208.
Fabbri, E., Miquel, C., Lucchini, V., Santini, A., Caniglia, R., Duchamp, C., Weber, J.M., Lequette, B., Marucco, F., Boitani, L., Fumagalli, L., Taberlet, P., Randi, E., 2007. From the Apennines to the Alps: colonization genetics of the naturally expanding Italian wolf (*Canis lupus*) population. Mol. Ecol. 16, 1661–1671.
Fabbri, E., Caniglia, R., Kusak, J., Galov, A., Gomercic, T., Arbanasic, H., Huber, D., Randi, E., 2014. Genetic structure of expanding wolf (*Canis lupus*) populations in Italy and Croatia, and the early steps of the recolonization of the Eastern Alps. Mamm. Biol. 79, 138–148.
Falush, D., Stephens, M., Pritchard, J.K., 2003. Inference of population structure from multilocus genotype data: linked loci and correlated allele frequencies. Genetics 164, 1567–1587.
Focardi, S., Montanaro, P., La Morgia, V., Riga, F., 2009. Piano d'azione nazionale per il capriolo italico (*Capreolus capreolus italicus*). Quad. Cons. Nat. 31, 1–172.
Fonseca, C., 2008. Winter habitat selection by wild boar *Sus scrofa* in southeastern Poland. Eur. J. Wildl. Res. 54, 361–366.
Fritts, S.H., Mech, L.D., 1981. Dynamics, movements, and feeding ecology of a newly protected wolf population in northwestern Minnesota. Wildl. Monogr. 80, 3–79.
Genovesi, P., 2002. Piano d'azione nazionale per la conservazione del lupo (*Canis lupus*). Quad. Cons. Nat. 13, 1–94.
Haber, G.C., 1996. Biological, conservation, and ethical implications of exploiting and controlling wolves. Conserv. Biol. 10, 1068–1081.
Hass, C.C., 2009. Competition and coexistence in sympatric bobcats and pumas. J. Zool. 278, 174–180.
Huggard, D.J., 1993. Prey selectivity of wolves in Banff National Park. I. Prey species. Can. J. Zool. 71, 130–139.
Iacolina, L., Scandura, M., Gazzola, A., Cappai, N., Capitani, C., Mattioli, L., Vercillo, F., Apollonio, M., 2010. Y-chromosome microsatellite variation in Italian wolves: a contribution to the study of wolf–dog hybridization patterns. Mamm. Biol. 75, 341–347.
Jędrzejewski, W., Niedzialkowska, M., Nowak, S., Jędrzejewska, B., 2004. Habitat variables associated with wolf (*Canis lupus*) distribution and abundance in northern Poland. Divers. Distrib. 10, 225–233.
Kaartinen, S., Luoto, M., Kojola, I., 2009. Carnivore–livestock conflicts: determinants of wolf (*Canis lupus*) depredation on sheep farms in Finland. Biodivers. Conserv. 18, 3503–3517.
Kernohan, B.J., Gitzen, R.A., Millspaugh, J.J., 2001. Analysis of animal space use and movments. In: Millspaugh, J.J., Marzluff, J.M. (Eds.), Radio Tracking and Animal Populations. Academic Press, San Diego, pp. 125–166.
Kovařík, P., Kutal, M., Machar, I., 2014. Sheep and wolves: is the occurrence of large predators a limiting factor for sheep grazing in the Czech Carpathians? J. Nat. Conserv. 22, 479–486.
Kruuk, H., Parish, T., 1981. Feeding specialization of the European badger *Meles meles* in Scotland. J. Anim. Ecol. 50, 773–788.
Landry, J.M., Olsson, P., Siegenthaler, A., Jackson, P., Farrell, A., 1999. The use of guard dogs in the Swiss Alps: a first analysis. KORA, Rep. n. 2.
Linnell, J.D., Odden, J., Smith, M.E., Aanes, R., Swenson, J.E., 1999. Large carnivores that kill livestock: do 'problem individuals' really exist? Wildl. Soc. Bull. 27, 698–705.
Linnell, J.D., Brøseth, H., Solberg, E.J., Brainerd, S.M., 2005. The origins of the southern Scandinavian wolf *Canis lupus* population: potential for natural immigration in relation to dispersal distances, geography and Baltic ice. Wildl. Biol. 11, 383–391.
Lovari, S., Sforzi, A., Scala, C., Fico, R., 2007. Mortality parameters of the wolf in Italy: does the wolf keep himself from the door? J. Zool. 272, 117–124.
Manly, B.F.J., Mcdonald, L.L., Thomas, D.L., Mcdonald, T.L., Erickson, W.P., 2002. Resource Selection by Animals: Statistical Design and Analysis for Field Studies. second ed. Kluwer Press, Dordrecht, Boston, London.
Marucco, F., McIntire, E.J.B., 2010. Predicting spatio-temporal recolonization of large carnivore populations and livestock depredation risk: wolves in the Italian Alps. J. Appl. Ecol. 47, 789–798.
Massolo, A., Meriggi, A., 1998. Factors affecting habitat occupancy by wolves in northern Apennines (northern Italy): a model of habitat suitability. Ecography 21, 97–107.
Mattioli, L., Apollonio, M., Mazzarone, V., Centofanti, E., 1995. Wolf food habits and wild ungulate availability in the Foreste Casentinesi National Park, Italy. Acta Theriol. 40, 387–402.
Mattioli, L., Capitani, C., Avanzinelli, E., Bertelli, I., Gazzola, A., Apollonio, M., 2004. Predation by wolves (*Canis lupus*) on roe deer (*Capreolus capreolus*) in north-eastern Apennine, Italy. J. Zool. 264, 249–258.
Mattioli, L., Capitani, C., Gazzola, A., Scandura, M., Apollonio, M., 2011. Prey selection and dietary response by wolves in a high-density multi-species ungulate community. Eur. J. Wildl. Res. 57, 909–922.
Mech, L.D., 1970. The Wolf: The Ecology and Behavior of an Endangered Species. The Natural History Press, New York, USA.
Mech, L.D., Boitani, L., 2003. Wolf social ecology. In: Mech, L.D., Boitani, L. (Eds.), Wolves: Behavior, Ecology, and Conservation. University of Chicago Press, Chicago, USA, pp. 1–34.
MEEDDAT–MAP, 2008. Plan d'action national sur le loup 2008–2012, dans le contexte français d'une activité importante et traditionnelle d'élevage. Ministère de l'Ecologie, de l'Energie, du Développement Durable et de l'Aménagement du Territoire et Ministère de l'Agriculture et de la Pêche, Paris, France.
Meriggi, A., Lovari, S., 1996. A review of wolf predation in southern Europe: does the wolf prefer wild prey to livestock? J. Appl. Ecol. 1561–1571.
Meriggi, A., Rosa, P., Brangi, A., Matteucci, C., 1991. Habitat use and diet of the wolf in northern Italy. Acta Theriol. 36, 141–151.
Meriggi, A., Brangi, A., Matteucci, C., Sacchi, O., 1996. The feeding habits of wolves in relation to large prey availability in northern Italy. Ecography 19, 287–295.
Meriggi, A., Brangi, A., Schenone, L., Signorelli, D., Milanesi, P., 2011. Changes of wolf (*Canis lupus*) diet in Italy in relation to the increase of wild ungulate abundance. Ethol. Ecol. Evol. 23, 195–210.
Meriggi, A., Dagradi, V., Dondina, O., Perversi, M., Milanesi, P., Lombardini, M., Raviglione, S., Repossi, A., 2015. Short term responses of wolves feeding habits to changes of wild and domestic ungulate abundance in Northern Italy. Ethol. Ecol. Evol. 27, 389–411.
Migli, D., Youlatos, D., Iliopoulos, Y., 2005. Winter food habits of wolves in Central Greece. J. Biol. Res. (Thessaloniki) 4, 217–220.
Milanesi, P., Meriggi, A., Merli, E., 2012. Selection of wild ungulates by wolves *Canis lupus* (L. 1758) in an area of the Northern Apennines (North Italy). Ethol. Ecol. Evol. 24, 81–96.
Miller, J.R.B., 2015. Mapping attack hotspots to mitigate human–carnivore conflict: approaches and applications of spatial predation risk modeling. Biodivers. Conserv. 24, 2887–2911.
Mills, L.S., Citta, J.J., Lair, K.P., Schwartz, M.K., Tallmon, D.A., 2000. Estimating animal abundance using noninvasive DNA sampling: promise and pitfalls. Ecol. Appl. 10, 283–294.
Okarma, H., 1995. The trophic ecology of wolves and their predatory role in ungulate communities of forest ecosystems in Europe. Acta Theriol. 40, 335–386.
Papageorgiou, N., Vlachos, C., Sfougaris, A., Tsachalidis, E., 1994. Status and diet of wolves in Greece. Acta Theriol. 39, 411–416.
Patalano, M., Lovari, S., 1993. Food habits and trophic niche overlap of the wolf *Canis lupus*, (L. 1758), and the red fox *Vulpes vulpes* (L. 1758) in a Mediterranean mountain area. Rev. Ecol. (Terre Vie) 48, 279–294.
Peterson, R.O., Ciucci, P., 2003. The wolf as a carnivore. In: Mech, L.D., Boitani, L. (Eds.), Wolves: Behavior, Ecology, and Conservation. University of Chicago Press, Chicago, USA, pp. 104–130.
Pires, A.M., Rodrigues, I.M., 2007. Multiple linear regression with some correlated errors: classical and robust methods. Stat. Med. 26, 2901–2918.
Poulle, M.L., Carles, L., Lequette, B., 1997. Significance of ungulates in the diet of recently settled wolves in the Mercantour Mountains (South Eastern France). Rev. Ecol. (Terre Vie) 52, 357–368.
Randi, E., 2008. Detecting hybridization between wild species and their domesticated relatives. Mol. Ecol. 17, 285–293.
Randi, E., 2011. Genetics and conservation of wolves *Canis lupus* in Europe. Mammal Rev. 41, 99–111.
Randi, E., Lucchini, V., Christensen, M.F., Mucci, N., Funk, S.M., Dolf, G., Loeschcke, V., 2000. Mitochondrial DNA variability in Italian and East European wolves: detecting the consequences of small population size and hybridization. Conserv. Biol. 14, 464–473.
Sand, H., Wikenros, C., Wabakken, P., Liberg, O., 2006. Effects of hunting on group size snow depth and age on the success of wolves hunting moose. Anim. Behav. 72, 781–789.
Schenone, L., Aristarchi, C., Meriggi, A., 2004. Ecologia del Lupo (*Canis lupus*) in Provincia di Genova: distribuzione, consistenza, alimentazione e impatto sulla zootecnia. *Hystrix* It. J. Mammal. 15, 13–30.
Seaman, D.E., Millspaugh, J.J., Kernohan, B.J., Brundige, G.C., Raedeke, K.J., Gitzen, R.A., 1999. Effects of sample size on kernel home range estimates. J. Wildl. Manag. 63, 739–747.
Sundqvist, A.K., Ellegren, H., Olivier, M., Vilà, C., 2001. Y chromosome haplotyping in Scandinavian wolves (*Canis lupus*) based on microsatellite markers. Mol. Ecol. 10, 1959–1966.
Teerink, B.J., 1991. Hair of West European Mammals: Atlas and Identification Key. Cambridge University Press, Cambridge, UK.
Thurber, J.M., Peterson, R.O., 1993. Effects of population density and pack size on the foraging ecology of gray wolves. J. Mammal. 74, 879–889.
Valière, N., Fumagalli, L., Gielly, L., Miquel, C., Lequette, B., Poulle, M.L., Weber, J.M., Arlettaz, R., Taberlet, P., 2003. Long-distance wolf recolonization of France and

Switzerland inferred from non-invasive genetic sampling over a period of 10 years. Anim. Conserv. 6, 83–92.
van Lière, D., Dwyer, C., Jordan, D., Premik-Banič, A., Valenčič, A., Kompan, D., Siard, N., 2013. Farm characteristics in Slovene wolf habitat related to attacks on sheep. Appl. Anim. Behav. Sci. 144, 46–56.
Vos, J., 2000. Food habits and livestock depredation of two Iberian wolf packs (*Canis lupus signatus*) in the north of Portugal. J. Zool. 251, 457–462.
Waits, L.P., Luikart, G., Taberlet, P., 2001. Estimating the probability of identity among genotypes in natural populations: cautions and guidelines. Mol. Ecol. 10, 249–256.
Wang, J., Santure, A.W., 2009. Parentage and sibship inference from multilocus genotype data under polygamy. Genetics 181, 1579–1594.
Wielgus, R.B., Peebles, K.A., 2014. Effects of wolf mortality on livestock depredations. PLoS One 9, 1–16.
Zimen, E., Boitani, L., 1975. Number and distribution of wolves in Italy. Z. Säugetierkd. 40, 102–112.
Zlatanova, D., Ahmed, A., Valasseva, A., Genov, P., 2014. Adaptive diet strategy of the wolf (*Canis lupus* L.) in Europe: a review. Acta Zool. Bulg. 66, 439–445.
Zuur, A.F., Ieno, E.N., Elphick, C.S., 2010. A protocol for data exploration to avoid common statistical problems. Methods Ecol. Evol. 1, 3–14.

View publication stats
